# AI and Wearables for Early Detection of Cognitive Impairment and Dementia: Systematic Review

**DOI:** 10.2196/86262

**Published:** 2026-02-23

**Authors:** Ander Cejudo, Markel Arrojo, Cristina Martín, Aitor Almeida

**Affiliations:** 1 Vicomtech Foundation, Basque Research and Technology Alliance (BRTA) Donostia-San Sebastián Spain; 2 Faculty of Engineering, University of Deusto Bilbao Spain; 3 Biodonostia Health Research Institute e-Health Group San Sebastián Spain

**Keywords:** actigraphy, artificial intelligence, circadian rhythms, cognitive impairment, dementia, machine learning, mHealth, mobile health, prevention, sleep, wearable devices

## Abstract

**Background:**

Traditional cognitive screening relies on episodic clinical assessments and may miss early changes preceding cognitive impairment and dementia. Wearable and mobile health technologies enable continuous monitoring of sleep, physical activity, and circadian rhythms, generating digital biomarkers that may support scalable early detection and prevention. However, current evidence remains fragmented across devices, analytic approaches, and cognitive outcomes.

**Objective:**

This study synthesizes and critically evaluates recent evidence on wearable devices for early detection and prevention of cognitive impairment and dementia, focusing on device categories, cognitive outcomes, analytic approaches, and prevention relevance.

**Methods:**

We searched PubMed, Scopus, ACM Digital Library, and SpringerLink for peer-reviewed studies published between January 2020 and December 1, 2025. Eligible studies included human participants with a mean age ≥50 years, continuous wearable-derived data collected for ≥24 hours, and validated cognitive outcomes; reviews, protocols, smartphone-only studies, and pharmacological interventions were excluded. Two reviewers independently screened studies, extracted data, and assessed risk of bias using the Appraisal Tool for Cross-Sectional Studies, Newcastle-Ottawa Scale, Cochrane Risk of Bias tool, and Quality Assessment of Diagnostic Accuracy Studies-2. Owing to substantial heterogeneity in devices, outcomes, and analytic methods, quantitative meta-analysis was not feasible; a structured narrative synthesis was conducted in accordance with PRISMA (Preferred Reporting Items for Systematic Reviews and Meta-Analyses) 2020 guidance. This study was not prospectively registered.

**Results:**

We included 49 studies, with sample sizes ranging from 14 to 91,948 participants (>200,000 total) and a median sample size of 145. Most used research-grade actigraphy (43/49, 87.8%), while fewer used commercial wearables (7/49, 14.3%). Cognitive outcomes most frequently relied on global screening instruments, including the Mini-Mental State Examination (18/49, 36.7%), followed by *ICD-10* (*International Statistical Classification of Diseases, Tenth Revision*)–based clinical diagnoses (7/49, 14.3%) and the Montreal Cognitive Assessment (7/49, 14.3%). Analytic approaches were predominantly statistical (36/49, 73.5%), with fewer studies applying machine learning (7/49, 14.3%) or deep learning methods (6/49, 12.2%). Statistical analyses linked disrupted sleep, circadian rhythm fragmentation, and irregular activity patterns to worse cognitive outcomes, with modest-to-moderate effect sizes. Machine learning and deep learning approaches reported classification performance with area under the curve values between approximately 0.70 and 0.95. Approximately one-quarter of the studies (13/49, 26.5%) addressed early detection or prevention through longitudinal risk estimation or predictive modeling. Key limitations included small sample sizes, short monitoring durations, and limited external validation.

**Conclusions:**

Wearable-derived behavioral markers show promise for early risk stratification. This review advances the field by shifting from descriptive associations toward a digital phenotyping framework evaluating artificial intelligence–driven prediction in the preclinical window. Unlike prior reviews focused on established dementia, it differentiates direct predictive evidence from indirect correlational findings and critically assesses methodological maturity. Continuous, passive monitoring may enable scalable detection of subtle behavioral changes, supporting earlier and more personalized risk reduction strategies.

## Introduction

### Background

Cognitive decline and dementia represent a major public health concern, with significant global and regional prevalence. In 2021, approximately 57 million people were affected by dementia worldwide, with more than 60% residing in low- and middle-income countries [1]. As the global population ages, dementia is expected to impact 78 million individuals by 2030 and 139 million by 2050 [2]. This increasing prevalence is attributed to both the rising number of older adults and the fact that dementia disproportionately affects the older population. Furthermore, the severity of the problem is anticipated to rise more sharply in regions with significant demographic changes, particularly low- and middle-income countries, where health care resources are often limited [3].

Dementia severely affects the quality of life, as it is a leading cause of disability and dependency in older individuals [4]. In addition to the physical and cognitive impairments experienced by individuals, dementia imposes a heavy emotional and economic burden on families and caregivers. Caregivers, often family members, are responsible for a substantial amount of care, with an average of 5 hours of daily care and supervision per individual affected. The global cost of dementia is estimated to exceed US $1.3 trillion, with informal caregiving accounting for approximately half of this expenditure. Health care and social systems face immense strain in responding to the needs of individuals with dementia, highlighting the urgent need for preventive measures and early intervention [2].

There are several modifiable risk factors for cognitive decline and dementia, including physical inactivity, unhealthy diets, smoking, and cardiovascular conditions such as hypertension, diabetes, and obesity [5]. Early identification of cognitive decline is crucial for enabling timely interventions. Traditional screening methods, such as the Mini-Mental State Examination (MMSE) and Montreal Cognitive Assessment (MoCA) [6], have limitations due to their episodic nature and reliance on periodic visits to health care providers. These methods are often influenced by situational factors [7,8] and may not detect early signs of decline or track changes in cognitive function over time [9,10]. In addition, performance on these instruments can be affected by educational level, language, and cultural background, and they provide only a cross-sectional snapshot of cognitive status [11]. Therefore, there is a growing need for continuous and more sensitive methods of monitoring cognitive health [12]. At the same time, the use of artificial intelligence (AI)–driven wearable technologies for continuous cognitive monitoring raises ethical and data privacy considerations, as such systems may involve the collection of sensitive behavioral and health-related data, underscoring the importance of appropriate data governance and responsible implementation [13].

Wearable devices present significant potential for the continuous and passive monitoring of key physiological variables, including activity levels, sleep patterns, heart rate, and gait. These devices can detect subtle, longitudinal changes in behavior that may occur before clinical symptoms emerge [14], allowing for the early identification of cognitive decline. AI enhances this process by analyzing data from wearables to identify patterns that indicate potential risks. AI algorithms can process large volumes of data, detecting even small shifts in physiological and behavioral patterns, which can inform personalized interventions. By leveraging AI for predictive analytics, it is possible to improve early detection capabilities, facilitating timely interventions and supporting more effective management of cognitive health [15]. This combination of wearables and AI offers a promising approach [16] to enhancing dementia prevention and care.

### Objective

Previous reviews have mainly examined associations between wearable-derived measures and dementia outcomes [16], often focusing on physiological or behavioral differences in individuals already diagnosed with the disease. Other reviews have emphasized specific domains, such as gait and mobility assessment using wearables [17] or the relationship between physical activity and cognitive decline [18]. In contrast, this review focuses on how wearable-derived data are analyzed, with particular attention to advanced analytic approaches, including machine learning and deep learning, used to support early detection and prevention across the cognitive aging spectrum, from subjective cognitive decline to clinical dementia. By synthesizing evidence within a digital phenotyping framework, this review highlights how longitudinal, multimodal wearable data combined with contemporary analytic methods can move beyond descriptive associations toward individualized risk characterization and prevention-oriented applications.

Thus, the main objective of this systematic review is to synthesize and critically evaluate the current evidence base and methodological maturity of wearable devices for the early detection and prevention of cognitive impairment and dementia. We structure our synthesis around 4 specific objectives:

- To evaluate the usage trends and application contexts of wearable technologies, specifically comparing the deployment of research-grade actigraphy vs consumer-grade devices.
- To synthesize the strength of statistical associations between wearable-derived digital biomarkers (eg, sleep and circadian rhythms) and standard clinical cognitive assessments.
- To critically assess the performance and robustness of analytic approaches, comparing the outcomes of conventional statistical modeling, machine learning, and deep learning techniques.
- To assess how early detection is addressed in wearable-based studies by differentiating between studies that directly implement early detection and those that frame their findings as potentially relevant to early detection.

By addressing these specific objectives, the review aims to clarify the current state of knowledge and identify priorities for advancing wearable-based approaches toward clinical and public health application.

## Methods

### Overview

The reporting of this systematic review was guided by the standards of the PRISMA (Preferred Reporting Items for Systematic Reviews and Meta-Analyses) 2020 statement [19] (Multimedia Appendix 1). The literature search strategy was developed and reported in accordance with the PRISMA-S (Preferred Reporting Items for Systematic Reviews and Meta-Analyses literature search extension) [20] to enhance transparency and reproducibility of the search process (Multimedia Appendix 2). The aim was to evaluate the association between wearable device–based sleep and activity measures and the onset or progression of cognitive impairment, including mild cognitive impairment (MCI), Alzheimer disease, and cognitive decline. The review process was carried out in multiple stages: identification of relevant articles, screening for eligibility, data extraction, and assessment of risk of bias. Studies that meet predefined inclusion criteria were included, and their findings were synthesized to provide insights into the role of wearable devices in monitoring cognitive health. No review protocol was prepared or registered before conducting this study.

### Information Sources and Search Strategy

A comprehensive search strategy was used across multiple electronic databases, including PubMed, Scopus, ACM Digital Library, and SpringerLink, to identify relevant studies published after 2020. The search was conducted using a combination of MeSH (Medical Subject Headings) terms and keywords related to cognitive impairment, sleep and activity measures, wearable devices, and their associations with cognitive outcomes. Databases were searched individually using their native interfaces. Duplicate records were removed, and studies published before 2020 were discarded to align the scope of this systematic review with contemporary research questions, analytic frameworks, and the use of wearable devices representative of current practice in digital phenotyping and early detection and prevention of cognitive decline. The search strategy was designed to capture a broad range of studies, including randomized controlled trials, cohort studies, and observational studies, to provide a comprehensive overview of the existing evidence.

The search strategy integrates key concepts relevant to studies on the early detection of cognitive impairment and dementia using wearable devices that monitor activity and sleep. Table 1 lists the search terms, organized into categories capturing cognitive impairment, sleep and activity measurements, wearable devices, and their application in early detection. Within each group, similar terms were combined using the OR operator, and the 4 groups were combined using the AND operator. Conceptually, the search followed the structure: (HC_1_ OR HC_2_ OR ...) AND (Data_1_ OR Data_2_ OR ...) AND (Wearable_1_ OR Wearable_2_ OR ...) AND (EarlyDetection_1_ OR EarlyDetection_2_ OR ...). Full search queries are included in Multimedia Appendix 3.

Alzheimer disease is specifically included under dementia, as it accounts for 60%-70% of all dementia cases [1]. Data capture must include information on sleep, activity, or physiological signals. Wearable devices may be commercial (eg, smartwatches) or noncommercial (ad hoc or research-grade devices) and are typically worn on the body for extended periods. This review focuses on studies advancing early detection and prevention, excluding research that examines features of the condition without the potential to identify cognitive decline before clinical diagnosis or progression to a more advanced stage.

To consider an article in this study, at least 1 search term of each group must be present in either the title or the abstract. This ensures that the manuscript has a clear focus on continuous data collection on sleep, activity, or physiological signals and links these measurements to cognitive impairment outcomes. The search was limited to studies published after 2020 and in English to ensure accessibility to full-text articles.

No additional search methods were used, and no study registries were searched. No targeted website searching or manual browsing of conference proceedings was conducted. Study authors and experts were not contacted for additional data. No published search filters were used. Search strategies were developed specifically for this review and were not adapted from prior reviews. No search updates were performed after the initial search, and the search strategy was not peer reviewed. Reference lists of included studies were screened to identify additional relevant records.

**Table 1:** Groups of search terms and representative keywords used to identify studies in the systematic review, organized by health condition, data type, wearable technology, and early detection.

Group	Search terms
Health condition	Cognitive impairment, mild cognitive impairment, dementia, cognitive decline, Alzheimer, neurocognitive disorder, and memory impairment
Data	Sleep, sleep duration, sleep quality, circadian rhythm, rest-activity rhythm, physical activity, actigraphy, accelerometry, heart rate, heart rate variability, HRV, respiratory rate, body temperature, respiration, skin temperature, step count, steps, and distance
Wearable	Wearable, wearable device, wearable devices, wearable sensor, wearable sensors, body-worn sensor, body-worn sensors, fitness tracker, smartwatch, smart band, wristband, wristbands, accelerometers, consumer-grade wearable, actigraph, actigraphy, ambulatory monitor, and digital biomarker
Early detection	Risk factor, onset, association, predictor, correlation, early detection, screening, and prevention

### Eligibility Criteria

The inclusion and exclusion criteria that guided the selection of studies for this systematic review are presented in Textbox 1. The criteria are designed to ensure that the review captures only the most relevant, recent, and methodologically sound research.

For inclusion, studies must be published between 2020 and 2025 to reflect the most recent developments in the field. Only publications identified by the predefined search queries were considered. Eligible studies must present either new statistical outcomes or AI-based results that provide original contributions. Because the review focuses on prevention and early detection, manuscripts must directly address these topics. Furthermore, included studies were required to involve human participants with a mean age of 50 years or older, ensuring relevance to an aging population. Another important condition was the continuous collection of data from wearable devices for at least 24 hours, which allows for robust and objective measurements. Only peer-reviewed journal articles were considered, discarding gray literature and preprints.

Exclusion criteria further refine the selection. Publications not written in English or not available in full text, for example, when behind an inaccessible paywall, were excluded. Review articles, study protocols, and books were not considered to maintain a focus on original research. Retracted publications were excluded. In addition, studies were not eligible if they did not apply validated measures of cognitive impairment or dementia, focused on pharmacological interventions, relied exclusively on self-reported data or smartphone-based sensing, or included only healthy participants without relevant clinical characteristics. No minimum sample-size threshold was applied during study selection. As a result, included studies span a wide range of sample sizes, including small-sample investigations (eg, n<30).

**Inclusion and exclusion criteria applied in the systematic review.**

**Inclusion criteria:**

- Published between 2020 and 2025
- Identified by the search queries
- Articles presenting new statistical outcomes or artificial intelligence results
- Manuscripts closely related to prevention and early detection
- Human participants must be involved in the study with average age of 50 years or older
- Continuous data captured from wearable devices for 24 hours or more

**Exclusion criteria:**

- Publication not in English
- Publication behind a paywall that cannot be retrieved
- Review papers, protocols, and books
- Retracted publications
- Validated measures of cognitive impairment and dementia not included
- Focus on drug or pharmacological interventions
- Only used self-reported data or smartphone-based sensing
- Only included healthy participants

### Risk of Bias Assessment

The risk of bias in included studies was assessed using the Appraisal Tool for Cross-Sectional Studies, the Newcastle-Ottawa Scale for cohort studies, the Cochrane Risk of Bias tool for randomized controlled trials, and the Quality Assessment of Diagnostic Accuracy Studies-2 for diagnostic studies [21]. These instruments evaluate domains such as selection, performance, detection, attrition, and reporting bias, as well as other potential sources of bias. Each study was independently evaluated by 2 coauthors (AC and MA), with disagreements resolved through discussion or consultation with a third coauthor (CM). The overall risk of bias was classified as low, moderate, or high, and these assessments were considered when interpreting the results of the review.

### Study Selection and Data Collection Process

Search results from each database were exported as BibTeX files and managed in Zotero (Corporation for Digital Scholarship), then converted to comma-separated values for automated preprocessing using a custom Python script. This process harmonized metadata across databases, removed duplicates, applied publication year filters, and verified the presence of predefined search terms. Automated preprocessing was conducted by the first author and independently verified by the second author. The following data items were extracted from each eligible study: bibliographic details (author and year), study design, participant characteristics (sample size, age, and diagnosis), wearable device specifications (type, brand, and sensor modality), monitoring duration, cognitive assessment tools, analytic methods (statistical vs AI), and primary outcomes (statistical associations or predictive performance metrics). Study selection followed a 3-stage screening process, with independent title, abstract, and full-text reviews performed by the first and second authors. Discrepancies were resolved through consensus or adjudication by a third author. Eligible studies were synthesized using a structured narrative approach aligned with predefined review objectives, grouping studies by device type, cognitive outcomes, analytic methods, and prevention-oriented contributions.

Analytic approaches were classified according to the overall modeling paradigm rather than individual algorithms. Studies were categorized as AI-based when wearable-derived data were analyzed within predictive modeling frameworks emphasizing automated pattern learning, such as machine learning or deep learning methods, including regression-based models implemented within machine learning pipelines (eg, automated feature selection or cross-validation). In contrast, studies relying on predefined statistical models for inferential or association analyses were classified as statistical model–based approaches. In this review, “direct” evidence was defined for studies demonstrating individual-level early detection capability, including both cross-sectional screening or discriminative analyses and longitudinal or interventional designs enabling risk estimation or prevention. Cross-sectional studies were therefore considered direct when they demonstrated the ability to distinguish preclinical or prodromal disease states at the time of assessment.

### Synthesis Methods

Quantitative synthesis was planned only for outcomes estimating a common construct using comparable analytical objectives and metrics. When outcomes cannot be mapped to a shared estimand for early detection, quantitative pooling was not conducted. In such cases, results were synthesized using a structured narrative approach aligned with the predefined review objectives. This approach grouped studies according to analytic strategy, cognitive assessment, and wearable measurement characteristics, without applying formal qualitative synthesis methodologies.

## Results

### Study Selection

**Figure 1:**
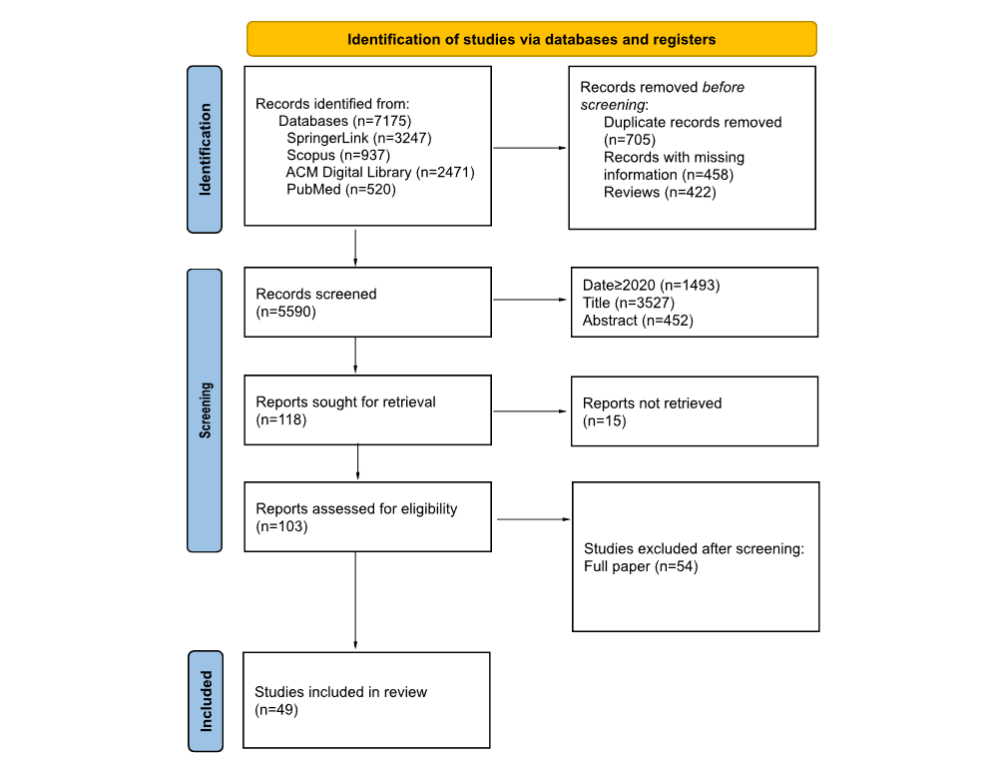
PRISMA (Preferred Reporting Items for Systematic Reviews and Meta-Analyses) flow diagram of the study selection process.

**Figure 2:**
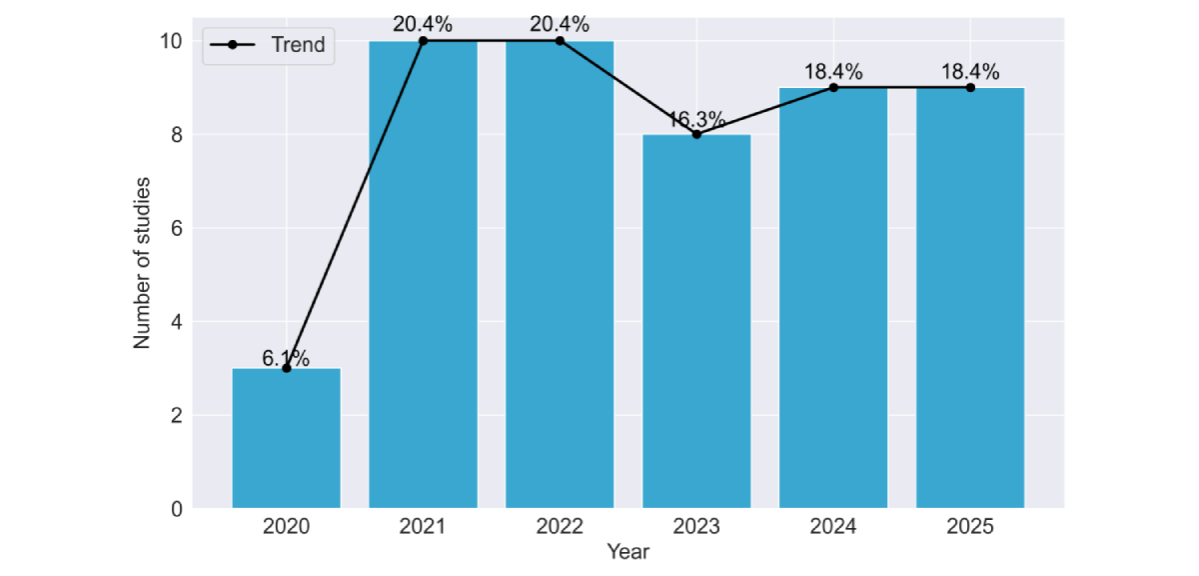
Temporal distribution of included studies by publication year (2020-2025). The bar chart and overlaid trend line illustrate the progression of research interest in the field. Data show a substantial increase in publication volume beginning in 2021, with research activity remaining consistently elevated through the 2024-2025 period. Data for 2025 are incomplete by 1 month.

The database search identified 7175 records (SpringerLink, n=3247; Scopus, n=937; ACM Digital Library, n=2471; and PubMed, n=520). After removal of 705 duplicates, 458 records with missing information, and 422 reviews, a total of 5590 records remained for screening. Of these, 1493 were excluded based on publication year (<2020, n=1493), title (n=3527), or abstract (n=452). The remaining 118 reports were sought for full-text retrieval, but 15 could not be obtained. A total of 103 full-text articles were assessed for eligibility, and 54 were excluded for not meeting the inclusion criteria. Finally, a total of 49 studies were included in this systematic review (Figure 1). Figure 2 shows the number of included studies by publication year. At the full-text screening stage, most studies were excluded because they did not include continuous wearable-based data collected over at least 24 hours, did not use wearable-derived data as part of the analysis, or did not report results relevant to the early detection of cognitive impairment or dementia. Table 2 shows the relevant information extracted from the included studies, and additional details are provided in Multimedia Appendix 4.

**Table 2:** Summary of included cross-sectional studies investigating wearable-derived measures in relation to dementia outcomes. The table reports study source, cross-sectional study design, participant groups and mean ages, and assessed risk of bias.

Source	Objective	Groups, n	Age (years), mean (SD)	Risk of bias
Lentzen et al [22]	Early detection	HC^a^=69; DEM^b^=160	69.2 (7.7)	Low
Jones et al [23]	Early detection	NCD^c^=21; MCI^d^=19	67.2 (8.4)	Low
Satomi et al [24]	Early detection	HC=25; MCI=63; DEM=21	79.3 (6.3)	Low
Sauers et al [25]	Risk assessment	HC=196; MCI=47	74	Low
Baril et al [26]	Risk assessment	HC=159; MCI=44	68.25	Low
Seo et al [27]	Early detection	HC=1519; MCI=353; DEM=87	70.1 (6.1)	Low
Huang et al [28]	Preventive intervention	HC=225; MCI=133; DEM=36	71.3(7.8); 72.9 (8.0)	Low
Palmer et al [29]	Progression monitoring	SCI^e^=29; MCI=79	68.8 (8.9)	Low
Lim [30]	Early detection	HC=14; DEM=4	≥65	Low
Boa Sorte Silva et al [31]	Early detection	MCI=113	73.1 (5.7)	Low
Kim et al [32]	Progression monitoring	HC=41; NCD=54	Median 60	Low
Basta et al [33]	General insight	MCI=40; DEM=49	78.3 (6.5)	Low
Roh et al [34]	Progression monitoring	MCI=70; DEM=30	Median 73	Low
Liu et al [35]	Early detection	HC=31; MCI=68	66.5	Low
Espinosa et al [36]	Early detection	SCI=44; MCI=99	67.4 (7.9)	Medium
Manning et al [37]	Risk assessment	HC=7; MCI=2; DEM=5	65.8	Medium
Ghosal et al [38]	Early detection	HC=54; DEM=38	73.4 (7.1)	Medium
Park et al [39]	Early detection	MCI/DEM=177; HC=14,482	74.1 (7); 39 (2.3)	Medium
Palmer et al [40]	Early detection	SCI=23; MCI=44	65.8 (7.9)	Medium
Wei et al [41]	Early detection	HC=20; DEM=8	Mean 59.2	Medium
Corbi and Burgos [42]	Early detection	HC=37; DEM=74	80.5	Medium

^a^HC: healthy controls.

^b^DEM: dementia.

^c^NCD: neurocognitive disorder.

^d^MCI: mild cognitive impairment.

^e^SCI: subjective cognitive impairment.

### Characteristics of Included Studies

A total of 49 studies were included in the review (summarized in Tables 3-8). Sample sizes ranged from 14 to 91,948 participants, with a median of 145. A total of 5 large cohorts heavily influenced the mean values (ranging between 47,371 and 91,948 participants). When stratified by analytic approach, studies using traditional statistical methods (36/49, 73.5%) had a median of 100 participants (mean 2597, SD 13,574), while those applying machine learning (7/49, 14.3%) had a median of 99 participants (mean 114.9, SD 76.4). Studies using deep learning (6/49, 12.2%) had a median of 108 participants (mean 397.6, SD 465.4), largely driven by population-scale datasets. Most studies were rated as low risk (34/49, 69.4%), nearly one-third (13/49, 26.5%) were classified as medium risk, and 2/49 (4.1%) as high risk. Participant populations were predominantly composed of individuals with MCI (29/49, 59.2%), dementia (35/49, 71.4%), or healthy controls (36/49, 73.5%). A smaller subset included participants with subjective cognitive decline (4/49, 8.2%) or other at-risk groups (2/49, 4.1%). Regarding study design, half of the studies were cross-sectional (21/49, 42.9%), followed by longitudinal cohorts (24/49, 49%), with fewer randomized controlled trials or diagnostic studies. Most studies (28/49, 57.1%) focused on early detection, while others assessed risk factors (8/49, 19.5%), monitored disease progression (7/49, 14.3%), or preventive interventions (5/49, 10.2%). Risk of bias (complete tables are provided in Multimedia Appendix 5) was generally low (35/49, 71.4%), though higher risk ratings were associated with small sample sizes and lack of external validation.

**Table 3:** Summary of included cohort studies investigating wearable-derived measures in relation to dementia outcomes. The table reports study source, study objective, cohort study design, participant groups and mean ages, and assessed risk of bias.

Source	Objective	Groups, n	Age (years), mean (SD)	Risk of bias
Baril et al [43]	Progression monitoring	HC^a^=159; MCI^b^=44	68.2 (5.4)	Low
Sun et al [44]	Early detection	HC=807; DEM^c^=270	80.9 (7.3)	Low
Winer et al [45]	Early detection	HC=82,377; DEM=452	62.0 (7.8)	Low
Haghayegh et al [46]	Early detection	HC=90,962; MCI or DEM=555	62.4 (7.8)	Low
Basta et al [47]	Progression monitoring	HC=29; MCI=49; DEM=32	80.3 (6.6)	Low
Milton et al [48]	Risk assessment	HC=476; MCI=164; DEM=93	82.5 (2.9)	Low
Skourti et al [49]	Progression monitoring	HC=146; MCI=231; DEM=128	72.8 (6.7)	Low
Gao et al [50]	Early detection	HC=409; MCI=61; DEM=81	58 (8)	Low
Jeon et al [51]	Early detection	HC=100; MCI=29	69.3 (7.7)	Low
Yi Lee et al [52]	Preventive intervention	HC=123; MCI=51	75.6 (6.9)	Low
Lysen et al [53]	General insight	HC=1262; DEM=60	66.1 (7.6)	Low
Agudelo et al [54]	Early detection	DEM=1035	55.2 (2.5)	Low
Ning et al [55]	Risk assessment	HC=91,212; DEM=736	Median 63	Low
Ning et al [56]	Risk assessment	HC=89,619; DEM=710	Median 64.9	Low
Zhao et al [57]	Risk assessment	HC=87,857; DEM=735	61.9 (7.9)	Low
Lu et al [58]	Early detection	HC=303; MCI=130; DEM=277	81.1 (5.2)	Low
Chan et al [59]	Early detection	HC=46,984; DEM=387	67.0 (4.0)	Low
Shi et al [60]	Early detection	HC=346; DEM=344	70.1 (6.9)	Low
Xiao et al [61]	Progression monitoring	HC=570; MCI=120; DEM=73	84.1	Low
Hoepel et al [62]	Preventive intervention	HC=1849; DEM=50	71.3 (9.26)	Low
Cho et al [63]	Early detection	DEM=222	80.4 (7.4)	Medium
Plotogea et al [64]	Early detection, preventive intervention	HC=25; SCI^d^=7; MCI=17; DEM=25	58.9 (9.8)	Medium
Targa et al [65]	Early detection	DEM=100	Median 76	Medium
Cho et al [66]	Risk assessment	DEM=145	81.2 (6)	Medium

^a^HC: healthy controls.

^b^MCI: mild cognitive impairment.

^c^DEM: dementia.

^d^SCI: subjective cognitive impairment.

**Table 4:** Summary of included studies with other study designs investigating wearable-derived measures in relation to dementia outcomes. The table reports study source, study objective (early detection or progression monitoring), study design (diagnostic study, randomized controlled trial, or quasi-experimental study), participant groups and ages, and assessed risk of bias.

Source	Objective	Study design	Groups, n	Age (years)	Risk of bias
Rykov et al [67]	Early detection	Quasi-experimental study	MCI^a^=17	60.3 (SD 4.5)	Medium
David et al [68]	Progression monitoring	Randomized controlled trial	DEM^b^=38	70 (SD 7)	Medium
Khosroazad et al [69]	Early detection	Diagnostic study	HC=45; MCI^c^=50	73.6	High
Hossain et al [70]	Early detection	Diagnostic study	HC=22; MCI=6; DEM=5	57.5	High

^a^HC: healthy controls.

^b^DEM: dementia.

^c^MCI: mild cognitive impairment.

**Table 5:** Wearable devices, recording parameters, and analytic methods used across the included studies. The table lists device type, monitoring duration, product and model, analytic approach (statistical, machine learning, deep learning, or clustering), primary task, and cognitive assessments applied.

Source	Wearable category	Wearable	Product (company name)	Duration (days), n	Model	Cognitive test
Espinosa et al [36]	Research	Actigraph	Actiwatch Spectrum (Philips)	13.7	Statistical	MMSE^a^
Lentzen et al [22]	Commercial research	Smartwatch	Fitbit Charge 3 (Google); AX3 (Axivity); Physilog (Gait Up)	56	Machine learning	Amsterdam IADL^b^
Manning et al [37]	Research	Actigraph	Actiwatch (Philips)	14	Statistical	MoCA^c^
Jones et al [23]	Commercial	Smartwatch	Apple Watch 8 (Apple)	6.9	Statistical	HVLT^d^
Satomi et al [24]	Research	Actigraph	ActTrust (Condor Instruments)	7	Statistical	CDR^e^
Baril et al [43]	Research	Actigraph	Actiwatch (Philips)	6.5	Statistical	RBANS^f^
Sauers et al [25]	Research	Actigraph	Sleep Profiler (Advanced Brain Monitoring); Actiwatch2 (Philips); Alice PDx (Philips)	6	Statistical	CDR
Sun et al [44]	Research	Actigraph	Actical (Philips)	14	Deep learning	NINCDS-ADRDA^g^
Winer et al [45]	Research	Accelerometer	AX3 (Axivity)	7	Statistical	TMT^h^
[67]	Multimodal research	Wrist-worn device	E4 wristband (Empatica)	10	Machine learning	NTB^i^
Haghayegh et al [46]	Research	Accelerometer	AX3 (Axivity)	6	Statistical	*ICD-10* ^j^
Basta et al [47]	Ad hoc	Actigraph	Not specified	7	Statistical	*ICD-10*
Baril et al [26]	Research	Actigraph	Actiwatch (Philips)	6.5	Statistical	MMSE
Milton et al [48]	Multimodal research	Actigraph	SleepWatch-O (Ambulatory Monitoring)	3	Statistical	MMSE
Cho et al [63]	Research	Actigraph	ActiGraph wGT3X-BT (ActiGraph)	14	Machine learning	MMSE
Seo et al [27]	Research	Accelerometer	HW-100 (Kao Corporation)	28	Statistical	MMSE
Huang et al [28]	Research	Actigraph	GENEActiv Original (Activinsights Company)	6	Statistical	MMSE
Khosroazad et al [69]	Research	Actigraph	Actiwatch (Philips)	7	Deep learning	HVLT
Skourti et al [49]	Research	Actigraph	Actigraph GT3XP (ActiGraph)	3	Statistical	RAVLT^k^
David et al [68]	Commercial	Smartwatch	Fitbit Charge 2 (Google)	13	Statistical	MoCA
Gao et al [50]	Research	Accelerometer	AX3 (Axivity)	7	Statistical	*ICD-10*
Ghosal et al [38]	Research	Accelerometer	Actigraph GT3x+ (ActiGraph)	7	Machine learning	CDR
Palmer et al [29]	Research	Actigraph	Actiwatch Spectrum (Philips)	10.5	Deep learning	MMSE
Jeon et al [51]	Research	Actigraph	Actiwatch 2 (Philips)	5.5	Statistical	MMSE
Lim et al [30]	Ad hoc	Smartwatch	Not specified	7	Deep learning	MMSE
Plotogea et al [64]	Research	Actigraph	Actiwatch Spectrum Pro (Philips)	7	Statistical	PHES^l^
Boa Sorte Silva et al [31]	Research	Actigraph	MotionWatch8 (CamNtech)	7	Statistical	MoCA
Park et al [39]	Commercial	Accelerometer	Fitmeter (FitNLife); ActiGraph AM-7164 (ActiGraph )	24.6 7	Deep learning	MMSE; CDR; GDS^m^
Palmer et al [40]	Research	Actigraph	Actiwatch Spectrum (Philips)	7	Statistical	MMSE
Kim et al [32]	Research	Actigraph	Spectrum Plus (Philips); Actiwatch Spectrum (Philips)	10.5	Statistical	NIH^n^
Targa et al [65]	Research	Actigraph	Actiwatch 2 (Philips)	14	Statistical	MMSE
Wei et al [41]	Commercial	Smartwatch	Mi Band 2 (Xiaomi)	14	Statistical	MMSE
Hossain et al [70]	Ad hoc	Smartwatch	Not specified	182.5	Machine learning	MMSE
Cho et al [66]	Ad hoc	Actigraph	wGT3X-BT (ActiGraph)	11.5	Statistical	MMSE
Corbi and Burgos [42]	Research	Accelerometer	AX3 (Axivity)	4	Machine learning	GDS
Basta et al [33]	Research	Actigraph	Actigraph GT3XP (ActiGraph)	3	Statistical	MMSE
Yi Lee et al [52]	Research	Actigraph	GENEActiv Original (ActivInsights)	7	Statistical	MoCA
Roh et al [34]	Commercial	Accelerometer	Fitmeter (FitNLife)	4	Statistical	SNSB^o^
Lysen et al [53]	Research	Actigraph	ActiWatch AW4 (CamNtech)	6	Statistical	MMSE
Agudelo et al [54]	Research	Actigraph	Actiwatch Spectrum (Philips)	7	Statistical	B-SEVLT-Sum^p^
Ning et al [55]	Research	Accelerometer	AX3 (Axivity)	7	Statistical	*ICD-10*
Ning et al [56]	Research	Accelerometer	AX3 (Axivity)	7	Statistical	*ICD-10*
Zhao et al [57]	Research	Accelerometer	AX3 (Axivity)	7	Statistical	*ICD-10*
Lu et al [58]	Research	Actigraph	wGT3X-BT (Ametris)	7	Statistical	MoCA
Chan et al [59]	Research	Smartwatch	Not specified	7	Statistical	*ICD-10*
Shi et al [60]	Research	Actigraph	wGT3X-BT (Ametris); Actiwatch Spectrum (Philips)	7; 3	Deep learning	MoCA
Liu et al [35]	Commercial	Accelerometer	W180 (Shenzhen Fitfaith)	14	Machine learning	MoCA
Xiao et al [61]	Research	Actigraph	Actigraph GT3XP (Ametris)	7	Statistical	TMT
Hoepel et al [62]	Research	Actigraph	GENEActiv Original (ActivInsights)	4	Statistical	MMSE

^a^MMSE: Mini-Mental State Examination.

^b^Amsterdam IADL: Amsterdam Instrumental Activities of Daily Living.

^c^MoCA: Montreal Cognitive Assessment.

^d^HVLT: Hopkins Verbal Learning Test-Revised.

^e^CDR: Clinical Dementia Rating.

^f^RBANS: Repeatable Battery for the Assessment of Neuropsychological Status.

^g^NINCDS-ADRDA: National Institute of Neurologic, Communicative Disorders and Stroke and Alzheimer’s Disease and Related Disorders Association.

^h^TMT: Trail-Making Test.

^i^NTB: Neuropsychological Test Battery.

^j^ICD-10: International Statistical Classification of Diseases, Tenth Revision.

^k^RAVLT: Rey Auditory Verbal Learning Test.

^l^PHES: Psychometric Hepatic Encephalopathy Score.

^m^GDS: Global Deterioration Scale.

^n^NIH: National Institutes of Health.

^o^SNSB: Seoul Neuropsychological Screening Battery.

^p^B-SEVLT-Sum: Brief Spanish-English Verbal Learning Test.

**Table 6:** Summary of machine learning used across studies, including model type, main performance metrics, analytical task (classification, regression, or clustering), and the corresponding feature selection or explainable artificial intelligence (XAI) techniques applied. Only the best-performing metric for each model is reported.

Source	Model	Metrics (best results)	Task	Feature selection/XAI
Lentzen et al [22]	LR^a^; DT^b^; RF^c^; XGBoost^d^	AUC^e^=0.73	Binary classification	SHAP^f^
Rykov et al [67]	LR; RF; XGBoost	*R*^2^=0.690; ρ=0.700; MAE^g^=0.460	Regression	Correlation-based feature selection
Cho et al [63]	LR; RF; GBM^h^; SVM^i^	AUC=0.929; accuracy=0.935; precision=0.800; sensitivity=0.956; *F*_1_-score (0.819)	Binary classification	Permutation feature importance
Ghosal et al [38]	GLM^j^; SOFR^k^; SOTDR^l^; SOTDR-L^m^	AUC=0.811; *R*^2^=0.333	Binary classification; regression	LASSO^n^/GEL^o^ penalties + functional coefficients
Hossain et al [70]	GBM; SVM; LR + LASSO; CTGAN^p^	Accuracy=0.948	Multiclass classification; regression	Correlation-based feature selection; wrapper feature selection
Corbi and Burgos [42]	Expectation-maximization clustering	Accuracy=0.910	Unsupervised clustering	Feature relevance via variable testing
Liu et al [35]	GBDT^q^; XGBoost^r^	Accuracy=0.757; recall=0.952; AUC=0.628	Binary classification	Permutation importance

^a^LR: logistic regression.

^b^DT: decision tree.

^c^RF: random forest.

^d^XGBoost: Extreme Gradient Boosting.

^e^AUC: area under the curve.

^f^SHAP: Shapley additive explanations.

^g^MAE: mean absolute error.

^h^GBM: gradient boosting machine.

^i^SVM: support vector machine.

^j^GLM: generalized linear model.

^k^SOFR: scalar-on-function regression.

^l^SOTDR: scalar on time-by-distribution regression.

^m^SOTDR-L: SOTDR via time varying L moments.

^n^LASSO: least absolute shrinkage and selection operator.

^o^GEL: group exponential LASSO.

^p^CTGAN: conditional tabular generative adversarial network.

^q^GBDT: gradient boosting decision tree.

^r^XGBoost: Extreme Gradient Boosting.

**Table 7:** Summary of deep learning used across studies, including model type, main performance metrics, analytical task (classification, regression, or survival analysis), and the corresponding feature selection or explainable artificial intelligence (XAI) techniques applied. Only the best-performing metric for each model is reported.

Source	Model	Metrics (best results)	Task	Feature selection/XAI
Sun et al [44]	CNN^a^; ElasticNet; RSF^b^	C-index=0.840; AUC^c^=0.880	Survival analysis (time to Alzheimer disease onset)	Gradient and Gini feature importance; hazard ratio interpretability
Khosroazad et al [69]	Neural network	AUC=0.880; sensitivity=0.870; specificity=0.890	Binary classification	Intrinsic via time-latency
Palmer et al [29]	MS-GAN^d^; Bayesian LR^e^	Dice=0.730; OR^f^=1.830	Segmentation; regression	Bayesian coefficients (odds ratio and CI)
Lim et al [30]	Neural network + PCA^g^	AUC=0.990	Binary classification	Correlation-based interpretability
Park et al [39]	1D convolutional autoencoder + LR	*R*^2^=0.979	Regression	Backward feature elimination
Shi et al [60]	CDPred^h^; XGBoost^i^	Hip: accuracy=0.84 and AUC=0.86; Wrist: accuracy=0.69 and AUC=0.73	Binary classification	Nonzero predictor relative importance ranking

^a^CNN: convolutional neural network.

^b^RSF: random survival forest.

^c^AUC: area under the curve.

^d^MS-GAN: multispecies generative adversarial network.

^e^LR: logistic regression.

^f^OR: odds ratio.

^g^PCA: principal component analysis.

^h^CDPred: cognitive decline predictor.

^i^XGBoost: Extreme Gradient Boosting.

**Table 8:** Summary of statistical approaches applied across studies, including model type, reporting metrics, covariates or adjustment factors, and main associations between wearable-derived variables and cognitive impairment or dementia indicators. Only the most relevant and statistically significant results for each study are presented. For wearable-derived metrics, arrows indicate the direction of association: an upward arrow (↑) denotes that higher values of the variable are associated with greater cognitive impairment, whereas a downward arrow (↓) indicates the opposite.

Source	Model	Metrics	Score
Espinosa et al [36]	General linear models	CWP^a^	IV^b^ (CWP<.001, ↑); IS^c^ (CWP<.001, ↓)
Manning et al [37]	Pearson correlation analysis	Pearson CC^d^, *P* value	Activity counts (CC=–0.829; *P*=.041, ↓)
Jones et al [23]	Linear regression models	*β* coefficient, 95% CI	IV (*β*≈.40, q=.022, ↑); sleep onset (*β*=–.28, 95% CI –0.55 to –0.02, ↑); IS (*β*=–.27, 95% CI –0.54 to 0.00, ↓)
Satomi et al [24]	Multinomial logistic regression	RR^e^, 95% CI, *P* value	IV (RR 3.21, 95% CI 1.40-7.34, *P*=.006, ↑); IS (RR 0.44, 95% CI 0.21-0.93, *P*=.03, ↓); M10 (RR 0.40, 95% CI 0.18-0.89, *P*=.02, ↓)
Baril et al [43]	Linear regression models	Standardized *β* coefficient, *P* value	Sleep duration (*β*=.384, *P*=.001, ↑)
Sauers et al [25]	Linear regression models	Estimate (*β*), *P* value	Sleep efficiency (*β*=–6.026, *P*<.001, ↓); sleep latency (*β*=11.302, *P*<.001, ↑); number of awakenings (*β*=6.585, *P*=.001, ↑)
Winer et al [45]	Cox proportional hazards models	HR^g^, 95% CI, *P* value	IS (HR 1.25, 95% CI 1.050-1.480; *P*=.007, ↑); amplitude (HR 0.79, 95% CI 0.650-0.960, *P*=.02, ↓); M10 (HR 0.75, 95% CI 0.610-0.940, *P*=.01, ↓); MESOR^h^ (HR 0.78, 95% CI 0.590-0.998, *P*=.048, ↓)
Haghayegh et al [46]	Cox proportional hazard models	HR, 95% CI	Amplitude (HR 1.32, 95% CI 1.17-1.49, ↑); M10 (HR 1.28, 95% CI 1.14-1.44, ↑); L5 (HR 1.15, 95% CI 1.10-1.21, ↑); IV (HR 1.14, 95% CI 1.05-1.24, ↑); rest-activity rhythm (HR 1.23, 95% CI 1.16-1.29, ↑)
Basta et al [47]	ANCOVA	*P* value	Sleep duration (night TST^j^, *P*<.001, ↑); TiB^k^ (night TiB, *P*<.001, ↑)
Baril et al [26]	Linear regression models	*P* value	Sleep duration (*P*<.05, ↑); activity counts (*P*<.05, ↑); circadian rhythm (*P*<.05, ↑)
Milton et al [48]	Multinomial logistic regression	OR^l^, 95% CI	Wake after sleep onset (OR 2.26, 95% CI 1.12-4.55, ↑); sleep efficiency (OR 2.15, 95% CI 1.03-4.47, ↓)
Seo et al [27]	Two-way ANCOVA	*P* value	Movement/acceleration (*P*=.03, ↓)
Huang et al [28]	Unconditional multivariable logistic regression	AOR^m^, 95% CI	MESOR (AOR 1.99, 95% CI 1.04-3.81, ↑)
Skourti et al [49]	Path models (analysis of moment structures)	Standardized *β* coefficient, *P* value	Sleep efficiency (direct *β*=.266, *P*=.001, ↓); wake after sleep onset (direct *β*=–.211, *P*=.001, ↑); TiB (24-hour TiB, indirect *β*=–.079, *P*<.001, ↑)
David et al [68]	Spearman rank correlation	Partial correlation coefficient (ρ), *P* value	Moderate-to-vigorous physical activity (ρ=0.558, *P*=.02, ↓)
Gao et al [50]	Cox proportional hazards	HR, 95% CI	Amplitude (HR 1.94, 95% CI 1.53-2.46, *P*<.001, ↑); IV (HR 1.49, 95% CI 1.18-1.88, *P*<.001, ↑); sleep duration (HR 1.28, 95% CI 1.06-1.55, *P*=.01, ↑)
Jeon et al [51]	Multivariate linear regressions	*β* coefficient, *P* value	Acrophase (*β*=–.256, *P*=.004, ↑)
Plotogea et al [64]	Multivariate logistic regression	OR, 95% CI, *P* value	Sleep efficiency (OR 0.803, 95% CI 0.711-0.904, *P*=.001, ↓); sleep latency (OR 1.212, 95% CI 1.063-1.383, *P*=.004, ↑)
Boa Sorte Silva et al [31]	Linear regression models	Unstandardized *β* (*β*), *P* value	Fragmentation index (*β*=.004, *P*=.046, ↑)
Palmer et al [40]	Fixel-wise linear regression; Bayesian multiple linear regression	*β* coefficient, *P* value	L5 (*β*=.29, *P*<.050, ↑)
Kim et al [32]	Multivariable linear models	Partial rank correlation (ρ), *P* value	IV (ρ=–0.44, *P*<.001, ↑); M10 (ρ=0.45, *P*<.001, ↓); IS (ρ=0.40, *P*=.009, ↓)
Targa et al [65]	Linear regression models	Effect size, *P* value	IV (effect size=–0.715, *P*=.013, ↑)
Wei et al [41]	Descriptive statistics; 1-tailed *t* test	Mean (SD), *P* value	Amplitude (0.93, SD 0.59, *P*=.030, ↓); IS (0.32, SD 0.19, *P*=.02, ↓); acrophase (44, SD 145, *P*<.001, ↓)
Cho et al [66]	Generalized linear mixed model	OR, 95% CI, *P* value	Sleep duration (OR 0.9, 95% CI 0.8-1.0, *P*<.001, ↓); activity counts (OR 0.02, 95% CI 0.0-0.5, *P*=.02, ↓)
Basta et al [33]	ANCOVA	Mean (SD), *P* value	Sleep duration (night TST=7.7-hour vs 7.2-hour, *P*=.011, ↑); sleep duration (24-hour TST=8.5-hour vs 7.8-hour, *P*=.012, ↑)
Yi Lee et al [52]	Multivariate logistic regression; multinomial logistic regression	AOR, 95% CI	Percent rhythm (AOR 0.26, 95% CI 0.08-0.79, ↓)
Roh et al [34]	Multiple linear regression	Estimate (*β*), SE, *P* value	MESOR (*β*=1.17, SE=0.37, *P*<.001, ↓); L5 (*β*=3.77, SE=1.22, *P*<.001, ↓)
Lysen et al [53]	Cox proportional hazards	HR, 95% CI	Sleep latency (HR 1.44, 95% CI 1.13-1.83, ↑); TiB (HR 1.40, 95% CI 1.04-1.88, ↑); sleep efficiency (HR 0.72, 95% CI 0.55-0.93, ↓); wake after sleep onset (HR 1.38, 95% CI 1.10-1.74, ↑)
Agudelo et al [54]	Survey linear regression models	*β* coefficient, *P* value	Sleep latency (*β*=–.003, *P*<.001, ↑); sleep duration (*β*=–.070, *P*<.05, ↑)
Ning et al [55]	Cox proportional hazards	HR, 95% CI	Moderate-to-vigorous physical activity (HR 0.69, CI 0.54-0.87, *P*<.001, ↓)
Ning et al [56]	Cox proportional hazards	HR, 95% CI	Moderate-to-vigorous physical activity (HR 0.60, 95% CI 0.40-0.90, ↓)
Zhao et al [57]	Cox proportional hazards	HR, 95% CI	Sleep duration (HR 0.801, 95% CI 0.717-0.893, ↓)
Lu et al [58]	Logistic regression models ANCOVA	OR, 95% CI	Relative amplitude (OR 1.68, 95% CI 1.12-2.50, ↑)
Chan et al [59]	Cox proportional hazards	HR, 95% CI	Bedtime (HR 1.52, 95% CI 1.22-1.85, ↑)
Xiao et al [61]	Cox proportional hazards	HR, 95% CI	MESOR (HR 2.45, 95% CI 1.52-3.94, ↓)
Hoepel et al [62]	Cox proportional hazards	HR, 95% CI	Sedentary behavior (HR 0.36, 95% CI 0.24-0.55, ↓)

^a^CWP: clusterwise *P* value.

^b^IV: intradaily variability.

^c^IS: interdaily stability.

^d^CC: correlation constant.

^e^RR: relative risk.

^f^M10: most active 10-hour.

^g^HR: hazard ratio.

^h^MESOR: midline estimated statistic of rhythm.

^i^L5: least active 5-hour.

^j^TST: total sleep time.

^k^TiB: time in bed.

^l^OR: odds ratio.

^m^AOR: adjusted odds ratio.

### Feasibility of Quantitative Synthesis

The feasibility of quantitative meta-analysis for early detection depends on whether the included studies estimate a common outcome construct or estimand. Quantitative synthesis requires studies to measure the same underlying construct. In the current literature, early detection is not operationalized as a single measurable outcome but is addressed through multiple, nonequivalent analytical objectives and metrics. The evidence base is characterized by the following dimensions:

Reported statistical metric: of the 49 included studies, 13 (26.5%) report performance metrics from AI-based approaches, such as accuracy or area under the curve. The remaining 36 studies report heterogeneous statistical effect measures, including hazard ratios for time-to-event analyses (n=16), odds ratios (ORs) for binary risk (n=8), and beta coefficients or correlation measures for continuous associations (n=12). These metrics reflect distinct inferential targets and are not interchangeable.

Device and sensor characteristics: most studies rely on research-grade actigraphy with access to raw accelerometry data (43/49, 87.8%), whereas a smaller subset uses consumer-grade wearables integrating additional sensors and proprietary processing (7/49, 14.3%). Differences in device type, sensor modality, and preprocessing lead to wearable-derived measures that do not map onto a common estimand.

Study design: study designs were divided between cross-sectional analyses assessing concurrent associations (21/49, 42.8%) and longitudinal cohort studies estimating prospective risk or disease onset (24/49, 49%). These designs address different research questions and operate over distinct temporal frameworks.

Clinical endpoint: cognitive outcomes included both categorical clinical diagnoses and continuous screening scores. The MMSE was the most frequently used outcome (18/49, 36.7%), followed by *ICD-10* (*International Statistical Classification of Diseases, Tenth Revision*)–based diagnoses and the MoCA (each 7/49, 14.3%). These endpoints differ in scale, sensitivity, and clinical interpretation.

Because early detection is not defined as a single measurable construct across studies, no common estimand can be specified for quantitative pooling. A structured narrative synthesis is therefore adopted to examine how analytical approaches, cognitive assessments, and wearable measurement strategies are used to demonstrate early detection capability across the cognitive continuum.

### Wearable Categories

Wearable devices were categorized into 4 groups according to their primary intended use. Research devices were by far the most common, reported in 87.8% (43/49) of studies, and included systems such as ActiGraph, Actiwatch, and accelerometer loggers (eg, Axivity AX3). These devices were purpose-built and validated for assessing activity, sleep, and circadian rhythms in research and clinical contexts. Commercial everyday wearables, such as Fitbit, Apple Watch, and Xiaomi Mi Band, were used in 14.3% (7/49) of studies; these multipurpose consumer devices are affordable and widely accessible but often provide limited access to raw data. Ad hoc prototypes, developed specifically for individual studies, accounted for 8.2% (4/49) of studies. Finally, multimodal research devices were used in 4.1% (2/49) of studies, typically integrating physiological sensing beyond accelerometry, such as heart rate, electrodermal activity, or peripheral arterial tonometry. Counts are nonexclusive because some studies used multiple device types in the same cohort [19].

Across all categories, wear time was typically about 1 week (median, 7 days), with durations ranging from 3-4 days to several months (up to 182.5 days in 1 longitudinal cohort). Most of the studies (29/49, 59.2%) relied on actigraphy devices to capture rest–activity rhythms and sleep, followed by accelerometer devices (12/49, 25.5%) and smartwatches (7/49, 14.3%).

### Cognitive Outcomes

**Figure 3:**
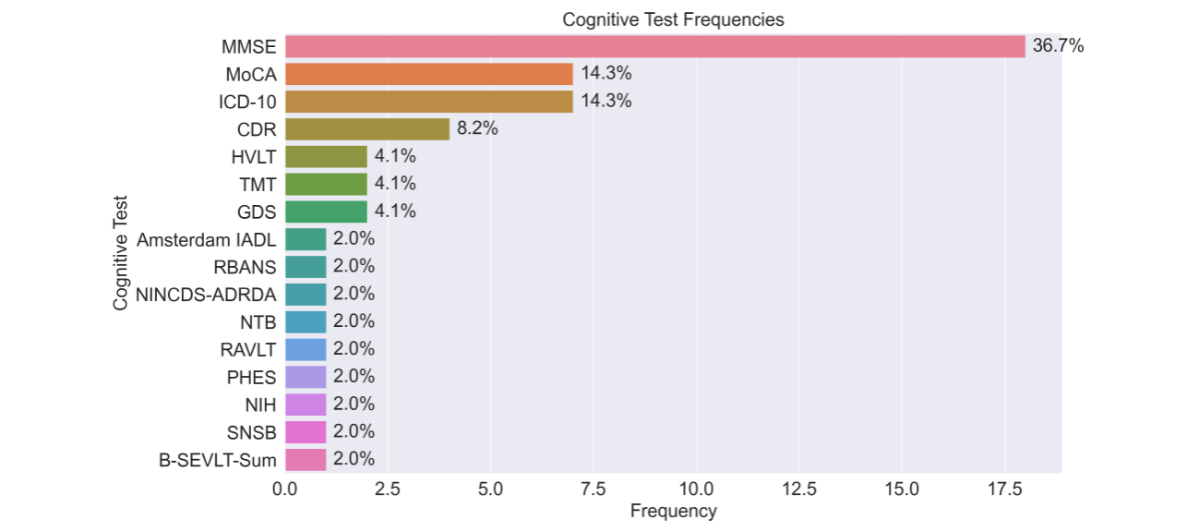
Frequency of cognitive assessment instruments across included studies. The bar chart illustrates the distribution of specific cognitive tests and diagnostic criteria used. Frequencies and percentages represent the prevalence of each tool within the total sample of studies. Amsterdam IADL: Amsterdam Instrumental Activities of Daily Living; B-SEVLT-Sum: Brief Spanish-English Verbal Learning Test; CDR: Clinical Dementia Rating; GDS: Global Deterioration Scale; HVLT: Hopkins Verbal Learning Test-Revised; ICD-10: International Classification of Diseases, Tenth Revision; MMSE: Mini-Mental State Examination; MoCA: Montreal Cognitive Assessment; NIH: National Institutes of Health; NINCDS-ADRDA: National Institute of Neurologic, Communicative Disorders and Stroke and Alzheimer’s Disease and Related Disorders Association; NTB: Neuropsychological Test Battery; PHES: Psychometric Hepatic Encephalopathy Score; RAVLT: Rey Auditory Verbal Learning Test; RBANS: Repeatable Battery for the Assessment of Neuropsychological Status; SNSB: Seoul Neuropsychological Screening Battery; TMT: Trail-Making Test.

The most frequently used cognitive measures were global screening tests, with their distribution shown in Figure 3. The MMSE was the most frequently used measure, applied in 36.7% (18/49) of studies. Clinical diagnoses based on *ICD-10* dementia or MCI codes were reported in 14.3% (7/49) of studies. The MoCA appeared in 14.3% (7/49) of studies and Clinical Dementia Rating was used in 8.2% (7/49) of studies. The Hopkins Verbal Learning Test and the Global Deterioration Scale were each used in 4.1% (2/49) of studies. A wide range of other neuropsychological and functional instruments, including the Amsterdam Instrumental Activities of Daily Living scale, Repeatable Battery for the Assessment of Neuropsychological Status, National Institute of Neurologic, Communicative Disorders and Stroke and Alzheimer’s Disease and Related Disorders Association criteria, Trail Making Test, Neuropsychological Test Battery, Rey Auditory Verbal Learning Test, Psychometric Hepatic Encephalopathy Score, National Institutes of Health toolbox, Seoul Neuropsychological Screening Battery, and Brief Spanish-English Verbal Learning Test-Sum, were each applied in 2% (1/49) of studies. The overall distribution of cognitive measures is shown in Figure 3.

One study applied AI-based approaches to directly predict cognitive test scores [67], while others relied on predefined cut-off points for classification [23,36,41,46,55,57]. Some studies used both direct score estimation and classification approaches [43,54]. A separate line of work focused on predicting survival outcomes, which were not directly comparable to cognitive test scores [43]. Finally, 2 studies addressed related regression tasks by estimating white matter characteristics in the brain from wearable-derived data [44,49].

**Figure 4:**
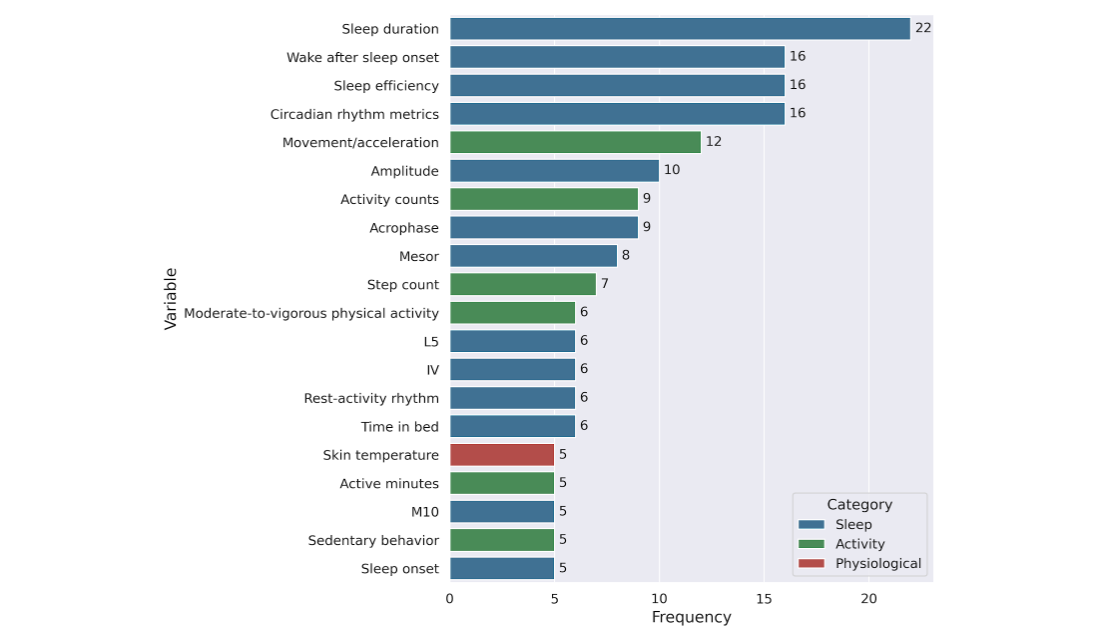
Top 20 most frequently used wearable-derived variables across studies. Variables are grouped by domain: sleep (blue), activity (green), and physiological (red). Frequencies represent the number of studies reporting each variable after standardization and filtering for wearable compatibility.

Across the 49 studies, the most frequently reported wearable-derived variables (complete list is provided in Multimedia Appendix 6) used to estimate cognitive assessments (Figure 4) were predominantly sleep-related. Sleep duration was the most frequently reported measure (22/49, 44.9%), followed by wake after sleep onset (16/49, 32.7%), sleep efficiency (16/49, 32.7%), and circadian rhythm metrics (16/49, 32.7%). Activity-related variables were reported less often overall, including movement/acceleration (12/49, 24.5%), activity counts (9/49, 18.4%), step count (7/49, 14.3%), moderate-to-vigorous physical activity (6/49, 12.2%), active minutes (5/49, 10.2%), and sedentary behavior (5/49, 10.2%). Physiological variables were least commonly reported, with skin temperature appearing in a small subset of studies (5/49, 10.2%).

### Wearable Data Analysis Approaches

Traditional statistical methods were the most frequently applied, reported in 73.5% (36/49) of studies (Table 3). These included group comparisons, correlation analyses, and regression models evaluating associations between wearable-derived activity or sleep metrics and cognitive outcomes. Commonly reported features were total daily activity counts, sleep duration and efficiency, circadian rhythm indices (intradaily variability, interdaily stability, and relative amplitude), and timing markers such as acrophase or sleep midpoint. The list of wearable-derived features used across these studies, with a description per feature, is included in Multimedia Appendix 6.

Most studies relying on conventional statistical approaches reported significant associations between wearable-derived variables and cognitive outcomes, although the magnitude of reported effects varied with sample size. In smaller studies (n<100), standardized regression coefficients and odds ratios tended to be larger, typically ranging from *β*≈.35-.55 or OR 2.0-3.1 [24,29,43]. In larger cohort studies (n≥100), effect estimates were generally lower, around *β*≈.10-.25 or OR 1.3-1.8 [45,46,49,54].

According to established benchmarks for social and behavioral sciences, standardized coefficients are typically categorized as small (*β*≈.20), medium (*β*≈.50), or large (*β*≈.80) [71]. While the estimates from larger cohorts in this review fall within the small-to-moderate range, they must be interpreted within the specific context of cognitive research. Given that cognitive performance is a multifactorial outcome influenced by a broad array of genetic, environmental, and demographic variables, the observation of stable effect sizes between .10 and .25 is highly meaningful. Such values represent robust contributions to the variance that, despite their conservative magnitude, suggest that wearable-derived metrics are reliable markers of cognitive health at a population level. While most associations reached statistical significance (*P*<.05), only a limited number of studies reported fully adjusted models or external validation [72].

Machine learning methods were applied in 14.3% (7/49) of studies, most often for classification tasks such as distinguishing MCI or dementia from healthy controls. Machine learning models included logistic regression, decision trees, support vector machines, random forests, and gradient-boosting approaches such as Extreme Gradient Boosting (XGBoost). Deep learning approaches were reported in 12.2% (6/49) of studies and were typically applied in larger datasets, where models captured temporal patterns of activity or sleep. Deep learning architectures included neural networks, convolutional neural networks, generative adversarial network–based approaches, and autoencoders. In addition, a small number of studies (2/49, 4.1%) applied clustering techniques to identify subgroups with distinct rest–activity profiles.

Across machine learning and deep learning studies, reported performance metrics varied widely depending on task, cohort size, and study design. Classification performance ranged from moderate discrimination in large, pragmatic settings (eg, area under the receiver operating characteristic curve [AUROC]≈0.73; n=229; dementia=160 and healthy controls=69 [23]) to higher values in smaller or more constrained cohorts (eg, AUROC≈0.93; n=222; affective symptoms=126 and not affective symptoms=96 [63]). Deep learning models reported metrics such as a C-index of approximately 0.84 (n=1077; dementia=270 and healthy controls=807) for long-horizon prediction of Alzheimer disease onset [44] and *R*² values approaching 0.98 (n=14,659; dementia=177 and healthy controls=14,482) for regression tasks estimating brain structural characteristics from wearable-derived data [40]. Several high-performing models were trained on small or imbalanced datasets, including studies with limited case numbers or highly unequal case–control ratios [28,31,40,70].

In addition, machine learning approaches applied to wearable-derived features achieved high discriminative performance across multiple tasks. Reported results included classification accuracies up to 93.5% [63] (n=222; sleep and nighttime behaviors=81 and not sleep and nighttime behaviors=141) for identifying high-risk individuals, accuracies of approximately 94%-95% (n=33; dementia=5, MCI=6, and healthy controls=22) across multiple cognitive impairment levels [70], area-under-the-curve values ranging from approximately 0.73 (n=229; dementia=160 and healthy controls=69) [23] to 0.99 (n=18; dementia=4 and healthy controls=14) [31] for early or prodromal Alzheimer disease detection, sensitivities and specificities exceeding 85% (n=95; MCI=50 and healthy controls=45) [69] for distinguishing MCI from normal cognition, and C-index values up to 0.84 (n=1077; dementia=207 and healthy controls=807) [44] for long-horizon Alzheimer disease onset prediction.

Interpretability approaches were reported in a limited subset of machine learning and deep learning studies. Explicit explainability techniques were applied in 46.1% (6/13) of AI-based studies, including Shapley additive explanations values or permutation feature importance in 2 machine learning studies [23,49], functional coefficients in 1 statistical–machine learning hybrid approach [31], and Bayesian coefficient estimation in 1 deep learning framework [32]. One additional study reported feature relevance through statistical testing in a clustering-based approach [39]. The remaining AI-based studies relied on feature-selection heuristics or dimensionality reduction methods, such as correlation-based selection, wrapper methods, or principal component analysis, without reporting interpretable model outputs [33,35,67,70].

### Prevention-Oriented Findings

The included studies were divided into 2 categories according to the type of contribution reported. A total of 22.4% (11/49) of studies explicitly provided quantitative results relevant to the early detection or prevention of cognitive decline (direct evidence), defined as studies demonstrating or validating predictive or preventive applications. The remaining 38 (77.6%) studies contributed indirect evidence, identifying associations between wearable-derived features and cognitive outcomes that may inform, but do not yet constitute, preventive or predictive applications.

Among studies providing direct evidence (n=11), longitudinal or interventional designs were commonly used. Specifically, 45.5% (5/11) of studies used longitudinal follow-up or randomized designs, while 54.5% (6/11) of studies relied on cross-sectional analyses. Direct-evidence studies included randomized intervention trials reporting outcomes such as slower progression to dementia, improved gait speed, or increased adherence to preventive programs, as well as longitudinal cohort studies showing that disrupted rest–activity rhythms, sleep fragmentation, and physical activity patterns predicted incident dementia or MCI over follow-up periods extending up to 8-15 years. Several studies further demonstrated that wearable-derived motor activity features could forecast clinical Alzheimer disease onset, with reported C-index values ranging from approximately 0.80 to 0.84.

In contrast, studies contributing indirect evidence (n=38) more frequently adopted cross-sectional designs. Of these, 52.6% (20/38) of studies were cross-sectional, whereas 47.4% (18/38) of studies used longitudinal designs. These studies primarily focused on identifying associations between wearable-derived sleep, circadian rhythm, activity, or physiological measures and cognitive outcomes, including global cognitive scores, neuropsychological performance, biomarkers, or clinical diagnoses, rather than on prediction or intervention.

Across all included studies, longitudinal designs accounted for 44.9% (22/49) of studies, while 55.1% (27/49) of studies used cross-sectional designs. This distribution reflects differing study aims, with direct evidence studies more often incorporating longitudinal follow-up or intervention components, and indirect evidence studies emphasizing associative analyses.

Overall, wearable technologies contributed to prevention-oriented research both directly, by demonstrating predictive and early detection capabilities in longitudinal and interventional settings, and indirectly, by identifying behavioral and physiological markers associated with future cognitive decline. This dual contribution reflects the role of wearables as both measurement tools for preventive interventions and sources of early behavioral risk markers in dementia research.

## Discussion

### Principal Findings

This systematic review synthesized 49 studies published since 2020 on wearable devices for the early detection and prevention of cognitive impairment and dementia. The findings are presented in 4 dimensions corresponding to the subobjectives described earlier.

### Wearable Devices and Measurement Contexts

Research-grade actigraphy and accelerometry devices were used in most of the included studies (43/49, 87.8%), reflecting both a strong methodological emphasis on validated access to raw accelerometry data and the relative maturity of actigraphy-based approaches in this field [73,74]. Evidence from these studies was derived predominantly from observational designs, including cross-sectional and longitudinal cohorts, and was frequently assessed as having low risk of bias, particularly among cohort studies with large samples [48,53,60]. Across actigraphy-based studies, sample sizes were heterogeneous but predominantly large, with nearly four-fifths enrolling 100 participants or more, and large samples were strongly associated with low risk of bias. While research-grade devices enable robust investigation of behavioral markers associated with cognitive impairment, their limited scalability and accessibility may constrain broader clinical and public health deployment [75].

In contrast, consumer-grade wearable devices were comparatively underrepresented despite their widespread adoption and potential for large-scale, longitudinal monitoring. Evidence from consumer-wearable studies was derived predominantly from cross-sectional designs and exhibited greater heterogeneity in sample size and methodological quality, with smaller studies more frequently assessed as having moderate risk of bias, reflecting restricted access to raw data and reliance on proprietary signal processing [41,68,76]. However, consumer-wearable evidence also included a small number of large cross-sectional studies assessed as having low risk of bias, indicating that methodological rigor is driven more by study scale and design than by device class alone [22,34,35]. Taken together, these findings suggest that different wearable device classes capture complementary aspects of behavior and physiology rather than interchangeable measurements, supporting the potential value of multimodal integration or purpose-driven device selection aligned with specific research or clinical objectives [76].

### Wearable-Derived Features and Cognitive Outcomes

Across included studies, cognition was frequently assessed using brief global screening instruments or standardized clinical diagnostic classifications, reflecting a pragmatic choice aligned with clinical practice and the feasibility requirements of observational research. Evidence derived from commonly used cognitive measures was drawn primarily from cross-sectional and cohort designs and was frequently assessed as having low risk of bias, particularly in larger cohort studies [46,50,55,56]. While global cognitive measures facilitate standardized comparisons across heterogeneous cohorts, their limited sensitivity to subtle or domain-specific changes constrains interpretation of associations with wearable-derived features, especially in preclinical populations [77].

Wearable devices were not used to measure cognition directly but to capture continuous behavioral and physiological signals that were statistically associated with cognitive outcomes. Sleep-related and circadian rhythm measures were examined most frequently and were reported across both cross-sectional and longitudinal studies, predominantly in cohort designs with large samples, and were most often assessed as having low risk of bias when derived from validated actigraphy devices and predefined metrics [33,47,59,62]. In contrast, physiological signals beyond accelerometry were examined in only a small subset of studies, which used heterogeneous, primarily noncohort designs, often involved small to medium sample sizes, and showed higher proportions of moderate-to-high–risk-of-bias assessments [35,41] limiting the strength of inferences that can currently be drawn from these measures and highlighting priorities for future methodological development.

### Analytical Approaches and Methodological Considerations

Conventional statistical methods were the predominant analytical approach, used in 73.5% (36/49) of studies, reflecting their interpretability and familiarity in clinical and epidemiological research. These methods were applied mainly in cross-sectional and longitudinal observational designs and were frequently assessed as having low risk of bias, particularly in large cohort studies using multivariable or survival models [45,46,50,55-62]. Across these studies, associations between wearable-derived features and cognitive outcomes were generally modest and sensitive to covariate adjustment, especially in large and heterogeneous samples, underscoring the importance of adequate sample size, rigorous model specification, and replication for reliable inference.

Machine learning and deep learning approaches were applied in a smaller subset of studies (13/49, 26.5%) and were used primarily for classification or prediction tasks. These studies were frequently exploratory, relied on small or imbalanced samples, and were more often assessed as having moderate to high risk of bias, largely due to limited external validation and optimistic performance estimates [67,69,70]. Across AI-based studies, higher predictive performance was more commonly reported in cross-sectional or nonlongitudinal designs using constrained datasets, whereas applications in larger or longitudinal cohorts generally yielded more modest performance estimates but were more often assessed as having low or moderate risk of bias, reflecting improved robustness and generalizability [35,44]. Overall, this pattern suggests a trade-off between performance and methodological robustness, in which performance gains observed in small or exploratory samples may reflect overfitting or cohort-specific structure rather than generalizable predictive signal.

### Implications for Early Detection and Prevention

Evidence supporting early detection or prevention was derived from a limited subset of studies providing direct prevention evidence, which more often used cohort-based or predictive modeling designs, enrolled larger samples, and were predominantly assessed as having low risk of bias. Representative examples include large longitudinal or predictive studies demonstrating that wearable-derived behavioral measures, particularly sleep–wake organization and circadian regularity, can precede clinical cognitive impairment by several years [39,46,56]. These findings support the potential role of wearables in early risk stratification rather than post hoc characterization.

In contrast, most of the included studies contributed indirect prevention evidence and relied primarily on cross-sectional or association-focused designs. Although these studies were frequently assessed as having low to moderate risk of bias and consistently reported associations between wearable-derived markers and cognitive status, their typically limited temporal resolution and lack of predictive validation constrained their ability to establish preventive relevance [24,27,33,40]. As a result, their contribution to prevention remains inferential rather than actionable.

Overall, the current evidence supports wearable devices as tools for monitoring and early risk identification rather than as stand-alone preventive interventions [78]. Across both direct and indirect evidence, disrupted sleep–wake patterns and circadian irregularity consistently emerged as markers of elevated cognitive risk [33,39,55]. Translating these associations into effective prevention strategies will require confirmation in larger, longitudinal, and externally validated studies [78] that integrate wearable-based risk stratification with clinical decision-making and behavioral intervention frameworks.

### Limitations

Several limitations should be considered when interpreting these findings. The included studies were heterogeneous in design, target populations, device types, and outcome definitions. Devices ranged from consumer-grade wearables to research actigraphy and multimodal physiological instruments, and the metrics derived from them were inconsistently defined. Cognitive outcomes also varied: global screening tools such as the MMSE and MoCA were most frequent, but other studies used different neuropsychological tests, functional measures, or clinical diagnoses. This heterogeneity precluded formal meta-analysis and necessitated a structured narrative synthesis. We did not formally assess the certainty of evidence (eg, using Grading of Recommendations Assessment, Development, and Evaluation), given the methodological heterogeneity of included studies and the predominance of observational designs.

The evidence base is also constrained by small sample sizes and short monitoring periods. In total, 8.2% (4/49) of studies enrolled 30 participants or fewer, while an additional 24.5% (12/49) included between 30 and 100 participants, indicating that more than one-third of the evidence relies on small cohorts. Furthermore, 67.3% (33/49) of studies monitored participants for only 1 week or less, limiting the ability to capture long-term variability in activity, sleep, or circadian rhythms. Only 18.4% (9/49) of studies systematically assessed real-world feasibility, including adherence, comfort, or long-term usability. Moreover, external validation was performed in just 6.1% (3/49) of studies, restricting the generalizability of reported associations or predictive models beyond the original cohorts. Thus, a key limitation of the current evidence is the risk of overinterpretation. Most studies were cross-sectional or small cohorts, and only few have undergone external validation [29,39,63]. In several studies, high accuracy or AUROC values were reported in the context of small or imbalanced datasets, conditions that increase the risk of overfitting and optimism bias. As a result, these performance estimates should be interpreted cautiously in the absence of external validation or evaluation in independent cohorts. While models often report high accuracy, these findings may not generalize. Preventive claims remain speculative until tested in large prospective cohorts or intervention trials. In addition, research-grade devices typically provide access to raw, high-resolution data and validated measurement pipelines, whereas consumer devices often rely on proprietary algorithms and offer limited transparency, which may affect comparability across studies.

The risk of bias was present to varying degrees. Although most studies were rated as low risk (34/49, 69.4%), nearly one-third (13/49, 26.5%) were classified as medium risk, and 4.1% (2/49) as high risk. The high-risk studies exemplify common problems such as selective sampling, inadequate reference standards, and small or restricted populations, all of which likely led to overestimation of diagnostic performance and limited the generalizability of their findings. Publication bias is also possible, as studies reporting positive or novel results are more likely to be published. Together, these issues highlight that the field remains fragmented, with methodological and reporting inconsistencies that restrict the strength and reproducibility of current evidence.

Limitations of the review process itself must also be acknowledged. The restriction to English-language publications and peer-reviewed journals may have introduced language or publication bias, potentially excluding relevant gray literature or local studies. Additionally, while we searched for 4 major interdisciplinary databases, the exclusion of specialized clinical indices (eg, PsycINFO or Embase) could have resulted in the omission of some relevant records.

### Implications for Future Research and Practice

This review highlights several priorities for future research and practice. A key challenge is bridging the gap between research-grade actigraphy and consumer wearables. While actigraphy remains the most validated tool for assessing sleep, activity, and circadian rhythms, limited use of consumer devices constrains scalability [79]. Evidence suggests that some multisensor consumer wearables can achieve accuracy comparable to research-grade actigraphs under controlled conditions [80,81], but direct comparisons across diverse populations and real-world settings remain needed. Where sufficient accuracy and reliability are demonstrated, consumer wearables could support large-scale, longitudinal monitoring for research and preventive care [82].

Another priority is the expanded use of advanced analytics and digital phenotyping. Longitudinal wearable data enable modeling of dynamic behavioral and physiological changes, supporting early and personalized prevention. However, machine learning and deep learning methods remain underused, particularly sequence-based models such as recurrent neural networks, temporal convolutional models, and transformer-based approaches (eg, Informer and TimesFM) that are well suited for time-series health data [83]. In addition, large language model–based frameworks (eg, HealthLLM) offer opportunities to integrate wearable-derived time series with clinical and contextual information [84]. Progress in this area will depend on access to larger, well-annotated datasets and multicenter collaboration, as well as the incorporation of explainable AI methods to ensure interpretability and clinical trust [85].

Finally, more prevention-oriented research is needed. Only 2 randomized controlled trials tested wearable-assisted interventions, and a small subset of observational studies examined prevention-relevant behaviors. Yet prevention is central to dementia care given the absence of disease-modifying treatments. Wearables could play a dual role, both by detecting early signs of decline and by monitoring modifiable lifestyle factors such as sleep, circadian rhythms, and physical activity [86,87]. Positioning wearables within preventive frameworks could therefore be among the most clinically meaningful directions for future research.

From a translational perspective, ethical and regulatory considerations will be critical for the clinical adoption of AI-based dementia risk prediction. Issues such as data privacy, transparency of algorithms, potential psychological impact of early risk labeling, and compliance with regulatory frameworks must be carefully addressed. At present, research-grade wearable devices and analytically transparent models appear more suitable for clinical research settings, whereas many consumer-grade devices and advanced AI approaches remain primarily experimental. Further validation, standardization, and regulatory oversight will be necessary before these methods can be routinely integrated into clinical practice.

Overall, this review advances the field by framing the transition from descriptive statistics to a predictive digital phenotyping framework. Distinct from prior reviews limited to established dementia, it synthesizes evidence specifically for the preclinical window, distinguishing findings with direct predictive utility from those offering only indirect correlational insights. It contributes a critical assessment of methodological maturity, identifying the heavy reliance on research-grade devices and the lack of external validation as primary barriers to implementation. Ultimately, these findings suggest that shifting to continuous, passive monitoring offers a scalable method to detect subtle behavioral deviations, creating opportunities for earlier, personalized risk reduction strategies [88].

## Appendix

Multimedia Appendix 1 PRISMA 2020 checklist.

Multimedia Appendix 2 PRISMA-S checklist.

Multimedia Appendix 3 Exact search queries used across all databases to identify records included in this review.

Multimedia Appendix 4 Extended study characteristics and principal findings of all the studies included in this systematic review.

Multimedia Appendix 5 Risk of bias assessments of included studies using validated tools appropriate to study design.

Multimedia Appendix 6 List and definitions of sleep, circadian rhythm, physical activity, and physiological variables extracted from wearable devices across included studies.

## Abbreviations

AI: artificial intelligence
ICD-10: International Statistical Classification of Diseases, Tenth Revision
MCI: mild cognitive impairment
MeSH: Medical Subject Headings
MMSE: Mini-Mental State Examination
MoCA: Montreal Cognitive Assessment
OR: odds ratio
PRISMA: Preferred Reporting Items for Systematic Reviews and Meta-Analyses
PRISMA-S: Preferred Reporting Items for Systematic Reviews and Meta-Analyses literature search extension
XGBoost: Extreme Gradient Boosting

## Data Availability

No new primary data were generated in this systematic review. Data extracted from the included studies and the curated list of screened articles are available from the corresponding author upon reasonable request. The Python code used to support automated screening and study management is not publicly available, as it was developed for internal use; however, it can be shared upon reasonable request for purposes of methodological transparency.
