# Supplementary material for: AI and Wearables for Early Detection of Cognitive Impairment and Dementia: Systematic Review
Source: J Med Internet Res. 2026 Feb 23;28:e86262. doi: 10.2196/86262 (PMC12972689; doi:10.2196/86262)
Supplement: Multimedia Appendix 3 [file jmir_v28i1e86262_app3.pdf]

PUBMED

("cognitive impairment"[tiab] OR "mild cognitive impairment"[tiab] OR dementia[tiab] OR "cognitive decline"[tiab] OR Alzheimer[tiab] OR "neurocognitive disorder"[tiab] OR "memory impairment"[tiab]) AND (wearable[tiab] OR "wearable device"[tiab] OR "wearable devices"[tiab] OR "wearable sensor"[tiab] OR "wearable sensors"[tiab] OR "body-worn sensor"[tiab] OR "body-worn sensors"[tiab] OR "fitness tracker"[tiab] OR smartwatch[tiab] OR "smart band"[tiab] OR wristband[tiab] OR wristbands[tiab] OR accelerometer[tiab] OR accelerometers[tiab] OR actigraph[tiab] OR actigraphy[tiab] OR "ambulatory monitor"[tiab] OR "digital biomarker"[tiab]) AND (sleep[tiab] OR "sleep duration"[tiab] OR "sleep quality"[tiab] OR circadian[tiab] OR "rest-activity rhythm"[tiab] OR "physical activity"[tiab] OR actigraphy[tiab] OR accelerometry[tiab] OR "heart rate"[tiab] OR "heart rate variability"[tiab] OR HRV[tiab] OR respiration[tiab] OR "respiratory rate"[tiab] OR "body temperature"[tiab] OR "skin temperature"[tiab] OR "step count"[tiab] OR steps[tiab] OR distance[tiab]) AND ("risk factor"[tiab] OR onset[tiab] OR association[tiab] OR predictor[tiab] OR prediction[tiab] OR correlation[tiab] OR "early detection"[tiab] OR screening[tiab] OR prevention[tiab])

SPRINGER

("cognitive impairment" OR "mild cognitive impairment" OR dementia OR "cognitive decline" OR Alzheimer OR "neurocognitive disorder" OR "memory impairment")
AND
(sleep OR "sleep duration" OR "sleep quality" OR circadian OR "rest-activity rhythm" OR "physical activity" OR actigraphy OR accelerometry OR "heart rate" OR "heart rate variability" OR HRV OR respiration OR "respiratory rate" OR "body temperature" OR "skin temperature" OR "step count" OR steps OR distance)
AND
(wearable OR "wearable device" OR "wearable devices" OR "wearable sensor" OR "wearable sensors" OR "body-worn sensor" OR "body-worn sensors" OR "fitness tracker" OR smartwatch OR "smart band" OR wristband OR wristbands OR accelerometer OR accelerometers OR actigraph OR actigraphy OR "ambulatory monitor" OR "digital biomarker")
AND
("risk factor" OR onset OR association OR predictor OR prediction OR correlation OR "early detection" OR screening OR prevention)

SCOPUS

TITLE-ABS-KEY(("cognitive impairment" OR "mild cognitive impairment" OR dementia OR "cognitive decline" OR Alzheimer OR "neurocognitive disorder" OR "memory impairment") AND (wearable OR "wearable device" OR "wearable devices" OR "wearable sensor" OR "wearable sensors" OR "body-worn sensor" OR "body-worn sensors" OR "fitness tracker" OR smartwatch OR "smart band" OR wristband OR wristbands OR accelerometer OR accelerometers OR actigraph OR actigraphy OR "ambulatory monitor" OR "digital biomarker") AND (sleep OR "sleep duration" OR "sleep quality" OR circadian OR "rest-activity rhythm" OR "physical activity" OR actigraphy OR accelerometry OR "heart rate" OR "heart rate variability" OR HRV OR respiration OR "respiratory rate" OR "body temperature" OR "skin temperature" OR "step count" OR steps OR distance) AND ("risk factor" OR onset OR association OR predictor OR prediction OR correlation OR "early detection" OR screening OR prevention))

ACM

("cognitive impairment" OR "mild cognitive impairment" OR dementia OR "cognitive decline" OR Alzheimer OR "neurocognitive disorder" OR "memory impairment")
AND
(sleep OR "sleep duration" OR "sleep quality" OR circadian OR "rest-activity rhythm" OR "physical activity" OR actigraphy OR accelerometry OR "heart rate" OR "heart rate variability" OR HRV OR respiration OR "respiratory rate" OR "body temperature" OR "skin temperature" OR "step count" OR steps OR distance)
AND
(wearable OR "wearable device" OR "wearable devices" OR "wearable sensor" OR "wearable sensors" OR "body-worn sensor" OR "body-worn sensors" OR "fitness tracker" OR smartwatch OR "smart band" OR wristband OR wristbands OR accelerometer OR accelerometers OR actigraph OR actigraphy OR "ambulatory monitor" OR "digital biomarker")
AND
("risk factor" OR onset OR association OR predictor OR prediction OR correlation OR "early detection" OR screening OR prevention)
