# Supplementary material for: AI and Wearables for Early Detection of Cognitive Impairment and Dementia: Systematic Review
Source: J Med Internet Res. 2026 Feb 23;28:e86262. doi: 10.2196/86262 (PMC12972689; doi:10.2196/86262)
Supplement: Multimedia Appendix 5 [file jmir_v28i1e86262_app5.pdf]

Appendix 5. Risk of bias tables

Table S1. Quality assessment of the included studies using the Appraisal tool for Cross-Sectional Studies (AXIS). Green circles indicate a "Yes" response (criterion met), while red circles indicate a "No" response (criterion not met). The total score for each study is presented in the final column.

Study	I1	I2	I3	I4	I5	I6	I7	I8	I9	I10	I11	I12	I13	I14	I15	I16	I17	I18	I19	I20	Total
[22]	🟢	🟢	🟢	🟢	🟢	🟢	🔴	🟢	🟢	🟢	🟢	🟢	🟢	🔴	🟢	🟢	🟢	🟢	🔴	🟢	🟢 17
[23]	🟢	🟢	🔴	🟢	🟢	🔴	🔴	🟢	🟢	🟢	🟢	🟢	🟢	🔴	🟢	🟢	🟢	🟢	🔴	🟢	🟢 15
[24]	🟢	🟢	🔴	🟢	🔴	🔴	🔴	🟢	🟢	🟢	🟢	🟢	🟢	🔴	🟢	🟢	🟢	🟢	🟢	🟢	🟢 15
[25]	🟢	🟢	🔴	🟢	🟢	🟢	🔴	🟢	🟢	🟢	🟢	🟢	🟢	🔴	🟢	🟢	🟢	🟢	🔴	🟢	🟢 16
[26]	🟢	🟢	🔴	🟢	🟢	🟢	🔴	🟢	🟢	🟢	🟢	🟢	🟢	🔴	🟢	🟢	🟢	🟢	🟢	🟢	🟢 17
[27]	🟢	🟢	🔴	🟢	🟢	🟢	🔴	🟢	🟢	🟢	🟢	🟢	🟢	🔴	🟢	🟢	🟢	🟢	🔴	🟢	🟢 16
[28]	🟢	🟢	🔴	🟢	🟢	🟢	🟢	🟢	🟢	🟢	🟢	🟢	🔴	🔴	🟢	🟢	🟢	🟢	🟢	🟢	🟢 17
[29]	🟢	🟢	🔴	🟢	🔴	🔴	🔴	🟢	🟢	🟢	🟢	🟢	🟢	🔴	🟢	🟢	🟢	🟢	🟢	🟢	🟢 15
[30]	🟢	🟢	🔴	🟢	🔴	🔴	🔴	🟢	🟢	🟢	🟢	🟢	🟢	🔴	🟢	🟢	🟢	🟢	🟢	🟢	🟢 15
[31]	🟢	🟢	🔴	🟢	🔴	🔴	🔴	🟢	🟢	🟢	🟢	🟢	🟢	🔴	🟢	🟢	🟢	🟢	🟢	🟢	🟢 15
[32]	🟢	🟢	🔴	🟢	🔴	🔴	🔴	🟢	🟢	🟢	🟢	🟢	🟢	🔴	🟢	🟢	🟢	🟢	🟢	🟢	🟢 15
[33]	🟢	🟢	🔴	🟢	🟢	🟢	🟢	🟢	🟢	🟢	🟢	🟢	🟢	🟢	🟢	🟢	🟢	🟢	🟢	🟢	🟢 19
[34]	🟢	🟢	🔴	🟢	🔴	🔴	🔴	🟢	🟢	🟢	🟢	🟢	🟢	🔴	🟢	🟢	🟢	🟢	🟢	🟢	🟢 15
[35]	🟢	🟢	🔴	🟢	🟢	🟢	🔴	🟢	🟢	🟢	🟢	🟢	🟢	🔴	🟢	🟢	🟢	🟢	🟢	🟢	🟢 17
[36]	🟢	🟢	🔴	🟢	🟢	🔴	🔴	🟢	🟢	🟢	🟢	🟢	🔴	🔴	🟢	🟢	🟢	🟢	🔴	🟢	🟡 14
[37]	🟢	🟢	🔴	🟢	🔴	🔴	🔴	🟢	🟢	🟢	🟢	🟢	🔴	🔴	🟢	🟢	🟢	🟢	🟢	🟢	🟡 14
[38]	🟢	🟢	🔴	🟢	🔴	🔴	🔴	🟢	🟢	🟢	🟢	🟢	🔴	🔴	🟢	🟢	🟢	🟢	🟢	🟢	🟡 14
[39]	🟢	🟢	🔴	🟢	🔴	🔴	🔴	🟢	🟢	🟢	🟢	🟢	🟢	🔴	🟢	🟢	🟢	🟢	🔴	🟢	🟡 14
[40]	🟢	🟢	🔴	🟢	🔴	🔴	🔴	🟢	🟢	🟢	🟢	🟢	🔴	🔴	🟢	🟢	🟢	🟢	🟢	🟢	🟡 14
[41]	🟢	🟢	🔴	🟢	🟢	🔴	🔴	🟢	🔴	🟢	🟢	🟢	🔴	🔴	🟢	🟢	🟢	🟢	🟢	🟢	🟡 14
[42]	🟢	🟢	🔴	🟢	🔴	🔴	🔴	🟢	🟢	🔴	🟢	🟢	🔴	🔴	🟢	🟢	🟢	🟢	🟢	🟢	🟡 13

Legend of Items for the Appraisal of Cross-Sectional Studies (AXIS)

Item 1: Were the aims/objectives of the study clear?
Item 2: Was the study design appropriate for the stated aim(s)?
Item 3: Was the sample size justified?
Item 4: Was the target/reference population clearly defined?
Item 5: Was the sample frame taken from an appropriate population base so that it closely represented the target/reference population under investigation?
Item 6: Was the selection process likely to select subjects/participants that were representative of the target/reference population under investigation?
Item 7: Were measures undertaken to address and categorise non-responders?
Item 8: Were the risk factor and outcome variables measured appropriate to the aims of the study?
Item 9: Were the risk factor and outcome variables measured correctly using instruments/measurements that had been trialled, piloted or published previously?
Item 10: Is it clear what was used to determined statistical significance and/or precision estimates?
Item 11: Were the methods (including statistical methods) sufficiently described to enable them to be repeated?
Item 12: Were the basic data adequately described?
Item 13: Does the response rate raise concerns about non-response bias? (inverse punctuation)
Item 14: If appropriate, was information about non-responders described?
Item 15: Were the results internally consistent?
Item 16: Were the results presented for all the analyses described in the methods?
Item 17: Were the authors' discussions and conclusions justified by the results?
Item 18: Were the limitations of the study discussed?
Item 19: Were there any funding sources or conflicts of interest that may affect the authors' interpretation of the results? (inverse punctuation)
Item 20: Was ethical approval or consent of participants attained?

* *Note on Scoring:* For Items 13 and 19, a "No" response (represented in green) indicates a positive assessment of study quality, as it signifies the absence of bias or conflicts of interest.

Table S2: Quality assessment of the included cohort studies using the Newcastle-Ottawa Scale (NOS). Each study is evaluated across three categories: selection, comparability, and outcome. Green circles represent a star awarded (low risk of bias), while red circles indicate no star awarded (high risk of bias). The final column displays the total score (out of 9 stars) for each study.

Study	Selection				Comparability		Outcome			Total	
	Representative of the exposed cohort	Selection of external control	Ascertainment of exposure	Outcome of interest not present at the	Comparability of: Main factor	Comparability of: Additional factor	Assesment of outcomes	Sufficient follow-up time	Adequancy of follow-up		
[43]	🟢	🟢	🟢	🟢	🟢	🟢	🟢	🟢	🟢	🟢	9
[44]	🟢	🟢	🟢	🟢	🟢	🟢	🟢	🟢	🟢	🟢	9
[45]	🟢	🟢	🟢	🟢	🟢	🟢	🟢	🟢	🟢	🟢	9
[46]	🟢	🟢	🟢	🟢	🟢	🟢	🟢	🟢	🟢	🟢	9
[47]	🟢	🟢	🟢	🟢	🟢	🟢	🟢	🟢	🟢	🟢	9
[48]	🟢	🟢	🟢	🟢	🟢	🟢	🟢	🟢	🟢	🟢	9
[49]	🟢	🟢	🟢	🟢	🟢	🟢	🟢	🟢	🟢	🟢	9
[50]	🟢	🟢	🟢	🟢	🟢	🟢	🟢	🟢	🟢	🟢	9
[51]	🟢	🟢	🟢	🟢	🟢	🟢	🟢	🟢	🟢	🟢	9
[52]	🟢	🟢	🟢	🟢	🟢	🟢	🟢	🔴	🟢	🟢	8
[53]	🟢	🟢	🟢	🟢	🟢	🟢	🟢	🟢	🟢	🟢	9
[54]	🟢	🟢	🟢	🟢	🟢	🟢	🟢	🟢	🟢	🟢	9
[55]	🟢	🟢	🟢	🟢	🟢	🟢	🟢	🟢	🟢	🟢	9
[56]	🟢	🟢	🟢	🟢	🟢	🟢	🟢	🟢	🟢	🟢	9
[57]	🟢	🟢	🟢	🟢	🟢	🟢	🟢	🟢	🟢	🟢	9
[58]	🟢	🟢	🟢	🟢	🟢	🟢	🟢	🟢	🟢	🟢	9
[59]	🟢	🟢	🟢	🟢	🟢	🟢	🟢	🟢	🟢	🟢	9
[60]	🟢	🟢	🟢	🟢	🟢	🟢	🟢	🟢	🟢	🟢	9
[61]	🟢	🟢	🟢	🟢	🟢	🟢	🟢	🟢	🟢	🟢	9
[62]	🟢	🟢	🟢	🟢	🟢	🟢	🟢	🟢	🟢	🟢	9
[63]	🔴	🔴	🟢	🔴	🟢	🟢	🔴	🔴	🟢	🟡	4
[64]	🔴	🔴	🟢	🟢	🔴	🟢	🔴	🟢	🟢	🟡	5
[65]	🔴	🔴	🟢	🔴	🟢	🟢	🔴	🟢	🟢	🟡	5
[66]	🔴	🔴	🟢	🔴	🟢	🟢	🔴	🔴	🟢	🟡	4

Table S3: Risk of bias assessment for quasi-experimental studies using the ROBINS-I tool. Green circles represent a low risk of bias, yellow circles indicate a moderate risk, and red circles signify a high risk. The domains (D1–D7) evaluate bias from confounding to the selection of reported results.

Study	D1	D2	D3	D4	D5	D5	D6	D7	Total
[67]	🟡	🟢	🟢	🟢	🔴	🟡	🟢	🟢	🟡

Domains:
D1: Bias due to confounding.
D2: Bias due to selection of participants.
D3: Bias in classification of interventions.
D4: Bias due to deviantions from intended interventions.
D5: Bias due to missing data.
D6: Bias in measurement of outcomes,
D7: Bias in selection of reported result.

Table A4: Risk of bias assessment for randomized controlled trials (RCTs) using the Revised Cochrane Risk-of-Bias tool (RoB 2). The symbols represent low risk (green), some concerns (yellow), and high risk (red) for each domain (D1–D5). The final column indicates the overall risk of bias for the study.

Study	D1	D2	D3	D4	D5	Total
[68]	🟢	🟡	🔴	🟡	🟢	🟡

Domains:

D1: Bias arising from the randomization process.

D2: Bias due to deviations from intended intervention.

D3: Bias due to missing outcome data.

D4: Bias in measurement of the outcome.

D5: Bias in selection of the reported result.

Table A5: Risk of bias assessment for diagnostic accuracy studies using the QUADAS-2 tool. Green circles indicate a low risk of bias, while red circles signify a high risk. Each study is evaluated across four key domains (D1–D4) to ensure the validity of the diagnostic findings.

Study	D1	D2	D3	D4	Total
[69]	🔴	🔴	🟢	🔴	🔴
[70]	🔴	🔴	🔴	🟢	🔴

Domains:
D1: Bias due to patient selection.
D2: Bias due to index test.
D3: Bias due to reference standard.
D4: Bias due to flow and timing.
