# Supplementary material for: AI and Wearables for Early Detection of Cognitive Impairment and Dementia: Systematic Review
Source: J Med Internet Res. 2026 Feb 23;28:e86262. doi: 10.2196/86262 (PMC12972689; doi:10.2196/86262)
Supplement: Multimedia Appendix 6 [file jmir_v28i1e86262_app6.pdf]

Variable	Category	Description
sleep duration	Sleep	Total sleep time per night.
sleep efficiency	Sleep	Percentage of time in bed spent asleep, estimated from actigraphy or multisensor data.
sleep latency	Sleep	Time taken to fall asleep after going to bed, inferred from inactivity onset and HR slowing.
wake after sleep onset	Sleep	Minutes awake after initially falling asleep.
sleep fragmentation	Sleep	Measure of disrupted sleep derived from frequency of awakenings.
time in bed	Sleep	Total duration spent in bed.
sleep onset	Sleep	Time when the transition from wake to sleep occurs.
number of awakenings	Sleep	Count of awakenings during the night.
circadian rhythm metrics	Sleep	Indicators of circadian regularity/fragmentation (e.g., interdaily stability, intradaily variability).
rest-activity rhythm	Sleep	Pattern of activity across day/night.
acrophase	Sleep	Clock time of peak circadian activity, estimated via cosinor analysis of actigraphy data.
mesor	Sleep	Midline estimating statistic of rhythm; mean level around which circadian rhythm oscillates.
amplitude	Sleep	Difference between rhythm peak and MESOR, reflecting strength of day/night contrast.
l5	Sleep	Average activity during the 5 consecutive hours of least activity within 24h (nighttime rest indicator).
m10	Sleep	Average activity during the 10 consecutive hours of highest activity within 24h (daytime activity indicator).
iv	Sleep	Intradaily variability; quantifies fragmentation of daily rhythm (higher = more irregular).
interdaily stability (is)	Sleep	Quantifies the stability of the 24-hour rest–activity rhythm across days (higher = more regular rhythm), derived from actigraphy time series.
percent rhythm	Sleep	Percentage of variance in activity explained by circadian rhythm model (higher = stronger rhythm).
step count	Activity	Total number of steps detected from accelerometer motion patterns.
activity counts	Activity	Aggregated index of activity intensity from accelerometer data.
movement/acceleration	Activity	Raw or processed accelerometer signal reflecting general body motion intensity.
moderate-to-vigorous physical activity	Activity	Time spent in higher-intensity activity based on accelerometer thresholds.
sedentary behavior	Activity	Time spent inactive (sitting or lying), detected as low accelerometer activity.
active minutes	Activity	Total time spent above a movement threshold, derived from accelerometer or HR zones.
heart rate	Physiological	Beats per minute measured via photoplethysmography (PPG) or ECG sensors.
heart rate variability	Physiological	Variation in intervals between heartbeats, indicating autonomic nervous system balance.

skin temperature	Physiological	Peripheral temperature measured via thermistors or infrared sensors.
electrodermal activity	Physiological	Skin conductance reflecting sweat gland activity (stress/arousal), measured by electrodes.
respiratory rate	Physiological	Breaths per minute estimated from chest motion, HR oscillations, or PPG amplitude.
oxygen saturation	Physiological	Percentage of oxygenated hemoglobin (SpO_2), measured via red/infrared PPG light.
blood pressure	Physiological	Estimated systolic and diastolic arterial pressure derived from PPG and pulse transit time.
